# Chemical profiling and clustering of various dried cannabis flowers revealed by volatilomics and chemometric processing

**DOI:** 10.1186/s42238-024-00252-w

**Published:** 2024-12-06

**Authors:** Pannipa Janta, Sornkanok Vimolmangkang

**Affiliations:** 1 Department of Pharmacognosy and Pharmaceutical Botany, Faculty of Pharmaceutical Sciences, Chulalongkorn University, Bangkok, 10330 Thailand; 2 Phyto Analytica Testing Laboratory, Leapdelab Co.,Ltd., Samut Prakan, 10130 Thailand; 3 Center of Excellence in Plant-Produced Pharmaceuticals, Faculty of Pharmaceutical Sciences, Chulalongkorn University, Bangkok, 10330 Thailand; 4 Research Cluster for Cannabis and its Natural Substances, Chulalongkorn University, Bangkok, 10330 Thailand

**Keywords:** Terpene profile, Cannabis aroma, GC-MS, HS-SPME, Discriminant marker

## Abstract

Cannabis flower scent is one of the key characteristics of the cannabis plant. The diverse scents impact user experiences and offer medicinal benefits. These scents originate from volatile compounds, particularly terpenes and terpenoids. This study characterized the volatile profile of 19 different dried cannabis flowers using gas chromatography-mass spectrometry coupled with headspace-solid phase microextraction (HS-SPME-GC-MS). A total of 75 compounds were identified, including alcohols, aldehydes, benzenes, esters, ketone, monoterpenes, monoterpenoids, sesquiterpenes, and sesquiterpenoids. Cluster analysis was able to group the 19 cannabis cultivars into five clusters based on volatile chemotypes using chemometric techniques of hierarchical cluster analysis (HCA) and principal component analysis (PCA). Potential discriminant markers of each cultivar were then analyzed using a supervised partial least squares discriminant analysis (PLS-DA) verified through Variable Importance in Projection values (VIP), identifying twenty discriminant markers. In addition, the correlations among 75 volatile compounds were also obtained. The findings of this study provide a valuable database of single cannabis cultivars, useful for identifying individual strains and verifying their quality. Clustering the cultivars by volatile chemotype can be used for the classification of cannabis in the market. The results of this study are expected to be a starting point for further cannabis breeding programs to expand knowledge of this plant. Furthermore, the proposed method is applicable to other aroma plants in the future.

**Supplementary Information:**

The online version contains supplementary material available at 10.1186/s42238-024-00252-w.

## Introduction

Recently, cannabis or Marijuana is one of the ancient plants popularly used for ingredients in several foods and beverages, fibers, and pharmaceutics (Radwan et al. 2017). This plant is also one of the globally significant agricultural crops, providing important features that affect psychotropic properties involving anxiety, paranoia, perceptual alteration, and cognitive deficits (Addo et al. 2021; Micalizzi et al. 2021). Moreover, the plant also contains various bioactive compounds providing antioxidant, antitumor, anti-inflammatory, antifungal, and antibacterial properties (Qamar et al. 2021). The main chemical components of cannabis are proteins, lipids, phytochemicals, minerals, pigments, flavonoids, terpenoids, cannabinoids, phytosterols, acids and alkaloids (Qamar et al. 2021). Among these components, cannabinoids and terpenoids are the main functional/medicinal constituents of cannabis which has been widely studied. As a result, regulations controlling the cultivation of cannabis have been adjusted in many countries worldwide, especially for medical purposes. Consequently, the global legal cannabis market has been growing steadily, estimated to be worth $57.18 billion in 2023 and expected to reach $147 billion by the end of 2027 (Langa et al. 2024).

The special focus is on the cannabis flower (also known as bud) which is the smokable, consumable, trichome-covered part of the female marijuana plant. This part is one of the selling points of the cannabis plant because it provides diverse scents including citrus, lemon, sweet, pungent, woody, earthy, and herbal (Gilbert and DiVerdi 2018) and also shows various medicinal benefits; for instance, treating pain and anxiety/depression (Vigil et al. 2023). The characteristic aroma of each cannabis flower especially resulted from terpenes (hydrocarbons) and terpenoids (oxygen-containing terpenes). Cannabis terpenes are varied since they can be changed according to their environmental and maturity conditions (Brown et al. 2019). More than 100 terpenes and terpenoids can be identified, which mostly accumulate in the glandular trichomes on the surface of the female inflorescences (Calvi et al. 2018).

Nowadays, the interest in many uses of cannabis flowers is increasing. Hundreds of cannabis cultivars are commercially available worldwide, particularly as a result of constant breeding and human selection (Langa et al. 2024), leading to alterations of the original plant. Moreover, various cannabis aromas can be rapidly developed to produce new scents, significantly impacting customers’ appreciation. Slight changes in cannabis aroma are hard to detect by direct human sniffing and difficult to quality control using biological methods. The different cannabis aromas mainly result from the variable compositions of volatile profiles. Hence, the characterization of the authentic volatile profile in each cannabis flower sample should be the primary focus. Recently, some cannabis flowers have disappeared from the market in Thailand, such as the Skunk Haze (SK) strain. However, it is still commercially available in online stores elsewhere. Notwithstanding, it cannot be guaranteed that it is the same SK strain. Thus, this presents a challenge for characterization, affecting laboratory testing, producers, and customers.

The suitable analytical technique used to study constituents of aroma compounds is gas chromatography-mass spectrometry (GC-MS) and higher separation performance: two-dimensional gas chromatography (GC×GC) (Franchina et al. 2020; Humston-Fulmer et al. 2020; Tungkijanansin et al. 2024). The benefit of an MS detector is precisely identifying volatile compounds in a sample by comparing their mass spectra with available libraries as well as accurate qualitative and quantitative analysis. Moreover, volatile compounds are often identified according to their retention index compared with the literature data (Girard 1996; Thongdorn-Ae et al. 2020; Janta et al. 2021a, b; Kakanopas et al. 2022). Sample preparation techniques that are conventionally applied for the extraction of volatile compounds is headspace solid phase microextraction (HS-SPME). This technique offers a simple and fast extraction process where volatile compounds in sample headspace can be adsorbed onto the SPME materials, e.g., divinylbenzene-based fibers for spice analysis and directly injected into the GC inlet (Vas and Vekey 2004).

Volatilomics is a subset of metabolomics based on the study of volatilome (biosynthesized volatiles) (Bicchi and Maffei 2012; Kasote et al. 2023). The qualitative and quantitative analysis of volatile compounds emitted by plants is included in volatilomics (Bicchi and Maffei 2012). The different volatile profiles and chemical markers lead to different aromas and medical properties (Gilbert and DiVerdi 2018; Gulluni et al. 2018; Kamal et al. 2018; Stith et al. 2020). Hence, this study aims to identify volatile profiles and marker compounds in 19 different dried cannabis flowers, covering the cultivars claimed for *C. sativa*, *C. indica*, and *C. hybrid.* Volatile profiles produced by individual cultivars were characterized by the basic conventional analytical method HS-SPME-GC-MS. The optimization of HS-SPME was performed and suitable conditions were applied for the extraction of volatile compounds in all samples. Chemometric tools were also employed for clustering cannabis aroma and identifying discriminant markers in each cannabis cultivar. To efficiently handle all of the data visualizations, both unsupervised (or clustering) and supervised classification methods (or discrimination) were applied, including hierarchical clustering analysis (HCA), principal component analysis (PCA), and partial least square discriminant analysis (PLS-DA). In addition, the correlations of volatile compounds were also studied using Pearson’s correlation coefficient. To the best of our knowledge, this is the first paper that studies volatile profiles with simple and green extraction in a large number of unmodified cannabis strains, covering three distinct groups; *C. sativa*, *C. indica*, and *C. hybrid*. The database of volatile profiles (referred to as fingerprinting) and clustering of cannabis aroma can be useful for the identification of both known and unknown single cannabis strains and serve as a determinant for quality control since it should consider not only morphology and cannabinoids but also the presence of terpenes (Ibrahim et al. 2019). Also, this study is expected to be a starting point for a dataset that can be used in breeding new cannabis cultivars to expand knowledge of volatile compounds. Moreover, this approach is expected to be applicable not only to cannabis strains but also to other aroma plants in the future.

## Materials and methods

### Dried cannabis flowers

The preparation of cannabis inflorescences involved drying and curing. After harvest, flowers were dried for 7–14 days in a dark, ventilated room at 60–70 °F (15–21 °C) and 55–65% humidity to preserve terpenes and cannabinoids. Trimming was done after drying (dry trimming). Once dried, flowers were cured in airtight containers for 2–3 weeks to enhance flavor, stabilize moisture, and improve quality. During the first week, containers were “burped” daily to release moisture and prevent mold, ensuring optimal storage conditions. In this study, 19 dried cannabis flower samples covering cultivars of *C. sativa*, *C. indica*, and *C. hybrid* were selected based on their different commercial data on feelings, aromas, and THC levels > 10%w/w as shown in Table S1 (Supplementary material). The samples were provided by an online store (Thailand), Medical Cannabis Center (Bangkok, Thailand), and Leapdelab Co., Ltd. (Samut Prakan, Thailand). *C. indica* samples were Skywalker OG (SW-OG), Purple Punch (PP), Wedding Cake (WC), White Widow (WW), Northern Light (NL), Grand Daddy Purple (GDP), Pure Michigan (PM), and Geta Fix (GF). *C. sativa* samples were Jack Herer (JH), Bruce Banner (BB), Green Crack Punch (GCP), Amnesia Haze (AH), Super Silver Haze (SH), Skunk Haze (SK), and Banana Gule (BG). *C. hybrid* included Frisian Duck (FD), Dulce de Fresa (DDF), and Critical Purple Kush (CPK). One sample of unknown origin was Hang Over G (HOG). The names of all cannabis strains are commercial names. All dried cannabis flowers were kept in a closed container and placed in a suitable area to avoid any degradation of volatiles before use.

### Chemical

A mixture of *n*-alkanes (C_7_-C_40_) purchased from Sigma Aldrich (St. Louis, MO) was used as a reference to calculate the linear retention index (LRI) of the compounds.

### Sample preparation

In this study, dried cannabis flower of SW-OG is a representative sample to study optimization of HS-SPME. To improve the extraction performance, each dried cannabis flower was ground by mortar to enhance the surface area before extraction (Atapattu and Johnson 2020). 0.1 g of ground flower was weighed and transferred into 20 mL glass vials closed with a 20 mm headspace aluminum cap with a sealed PTFE/silicone septum. The glass vial and headspace aluminum cap were purchased from Agilent Technologies Inc., US.

### HS-SPME

In this study, an SPME 50/30 µm DVB/CAR/PDMS fiber and holder purchased from Supelco (Sigma-Aldrich, Bellefonte, PA) were used to extract volatile compounds in the samples. Before the real sample analysis, the blank fiber was injected to check the background signal from the fiber. To avoid off-flavor effects and cannabinoid interference from the high temperatures of HS-SPME, the vials were heated in a water bath at the low temperature of 40 °C (Myers et al. 2021; Pachura et al. 2022; Mahattanatawee et al. 2005; Ma et al. 2013)The SPME fiber was then exposed inside the vial to extract volatile compounds in the sample’s headspace. Unless otherwise stated, the extraction time was 30 min. All samples were performed in triplicate.

### GC-MS

The determination of volatile compounds was performed using an Agilent 7890B gas chromatograph coupled with an Agilent 7000D mass spectrometer (Agilent Technologies Inc., US). Volatile compounds were separated on a DB-WAX capillary column (60 m × 0.25 mm i.d., 0.25 μm film thickness; J&W Scientific, USA) using high-purity helium as the carrier gas with a flow rate of 1 mL/min. Dried cannabis flowers were injected into the GC injection port at 250 °C. A linear temperature program from 60 to 250 °C with a ramp of 4 °C/min (total run time of 53 min) was assigned for the separation of volatile compounds and a split ratio of 1:5. The temperature of the ion source in the MS was set at 230 °C. The electron ionization voltage was set at −70 eV. The mass spectra were acquired over the mass range of 33–500 Da with a scan time of 200 ms.

### Data processing

The chromatographic peak and MS data of each sample were identified using Agilent MassHunter software. The data processing and presentation were performed using Microsoft Excel.

#### Compound identification

Separated compounds were tentatively identified by the comparison of their MS spectra with those obtained from the NIST 14 library. The identification criteria were selected with a match score of > 650 and a difference of 30 units (Janta et al. 2021a, b) between the calculated retention index (*I*) and the *I* data from the literature for the same (or a similar) stationary phase (Δ*I*). In this study, the DB-WAX capillary column, a polar stationary phase, was utilized. Thus, *I* literatures of the polar stationary phase were applied to calculate Δ*I*.

The experimental *I* value for each peak in the chromatograms relative to the alkane retention time data was obtained by injection of an alkane mixture under the same experimental conditions used for the sample separation. *I* values for the linear temperature-programmed separation were calculated according to the literature (Girard 1996; Bianchi et al. 2007).

$$\:\:\varvec{L}\varvec{R}\varvec{I}=100\varvec{n}+100\left(\frac{{\varvec{t}}_{\varvec{R}\left(\varvec{i}\right)}-{\varvec{t}}_{\varvec{R}\left(\varvec{n}\right)}}{{\varvec{t}}_{\varvec{R}(\varvec{n}+1)}-{\varvec{t}}_{\varvec{R}\left(\varvec{n}\right)}}\right)$$

where *t*_R_ is retention time of peak *i*. n and *n* + 1 are the carbon numbers of alkane standards bracketing the peak *i*.

#### Multivariate statistical analyses

In this study, R version 4.4.0 (R Core Team 2024) was employed to analyze the statistical evaluation of the volatile compounds in 19 dried cannabis flowers. The obtained data were presented in %area normalization calculated from an individual peak area divided by the total peak area of all identified compounds in each sample. All the samples were performed in 5 replicates (*n* = 5). Therefore, the covariance data were 95 × 75 matrices (95 samples × 75 individual compounds = 7,410 data points). The figures-of-merit of each sample, analyzed in 5 replicates, were evaluated by calculating %RSD of the average total peak area, average total peak height, and average peak width of all the volatile compounds detected. The results showed that %RSD of the average total peak area ranged from 2.1 to 11.0%, the average total peak height from 2.2 to 8.8%, and the average peak width from 0.8 to 6.9%. These values are presented in Table S2 (Supplementary material). Multivariate statistical analyses consist of hierarchical cluster analysis (HCA), principal component analysis (PCA), and partial least squares discriminant analysis (PLS-DA). HCA and PCA are the same class of unsupervised multivariate analysis techniques. HCA is commonly used to visualize the relationships within multivariate datasets. In this study, HCA was conducted using the hclust function in the stats package (R Core Team 2024), which was visualized using the ggdendro package (de Vries and Ripley 2024). PCA is used to visualize the overview of the correlation between samples and observed variables and show which compounds contribute different trends from each other. Finally, PCA is shown in a group of samples based on the class of observed volatiles. PCA was generated using the FactoMineR package (Lê, Josse, and Husson 2008). PLS-DA is a supervised statistical method commonly used in multivariate data analysis for predictive and descriptive modeling and discriminative variable selection. This technique is particularly beneficial for identifying biomarkers, distinguishing between physiological states, and predicting class membership for new samples based on their metabolite profiles. PLS-DA was performed using the MetaboAnalystR package (Chong et al. 2019). The correlations between volatile compounds were calculated using Pearson’s correlation (Zurr, 2009) and visualized in a heatmap using the ggcorrplot package.

## Results and discussion

### Optimization of HS-SPME extraction time

**Fig. 1:**
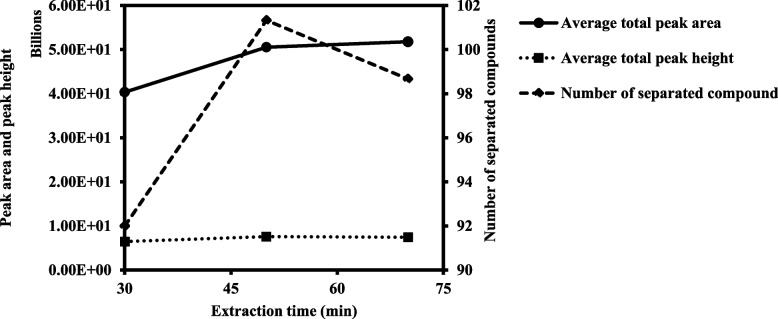
Average total peak area (solid line), average total peak height (dotted line), and average number of separated compounds (dashed line) of all the volatile compounds detected in the extracted Skywalker OG flower at various HS-SPME extraction times

The HS-SPME extraction time was optimized to achieve the best extraction with satisfactory retention times. The effect of the extraction time (30, 50, and 70 min) on the extraction efficiency was determined at 40 °C. The chromatographic parameters of average total peak area, average total peak height, average peak width, and average number of separated peaks of all the volatile compounds detected were determined. All conditions were analyzed in triplicates and the results were summarized in Table 1; Fig. 1.

**Table 1:** Average total peak area, average total peak height (dotted line), and average number of separated compounds (dashed line) of all the volatile compounds detected in the extracted SK OG flower at various HS-SPME extraction times (*n* = 3)

Extraction time (min)	Average total peak area ×10^10^(%RSD)	Average total peak height ×10^9^(%RSD)	Average number of separated compounds (%RSD)	Average peak width (%RSD)
30	4.04 (11.96)	6.45 (8.37)	92 (2.17)	0.19 (0.57)
50	5.05 (7.81)	7.59 (6.96)	101 (1.51)	0.19 (0.62)
70	5.18 (6.51)	7.44 (4.54)	99 (1.55)	0.19 (1.61)

According to Table 1; Fig. 1, the average total peak area gradually rose from 50 to 70 min. However, the average total peak height and average number of separated compounds slightly decreased as extraction time increased. The available spaces on fiber adsorbent material were filled with volatiles during a longer extraction time. However, once all sites on adsorbent material of fiber were completely occupied, the extraction efficiency would not increase and could even accelerate desorption feasibility. Therefore, prolonged extraction times are not suitable for some samples (Wei et al. 2021). Another consideration is that the extraction process should be performed concurrently with the GC-MS analysis of the previous sample to reduce waste time during extraction and analysis. Therefore, an HS-SPME extraction time of 50 min was selected to best fit the total GC-MS separation time (53 min), showing a better average total peak area and an average number of separated compounds.

### GC-MS analysis of dried cannabis flower and compound identification

The optimized HS-SPME extraction time of 50 min along with an extraction temperature of 40 °C was applied for all dried cannabis flowers. All compounds detected in the GC-MS chromatograms were identified according to a comparison of their mass spectra with those from the NIST 14 library with match scores of > 650 as well as experimental and literature values of the linear retention index (Δ*I* ± 30).

**Fig. 2:**
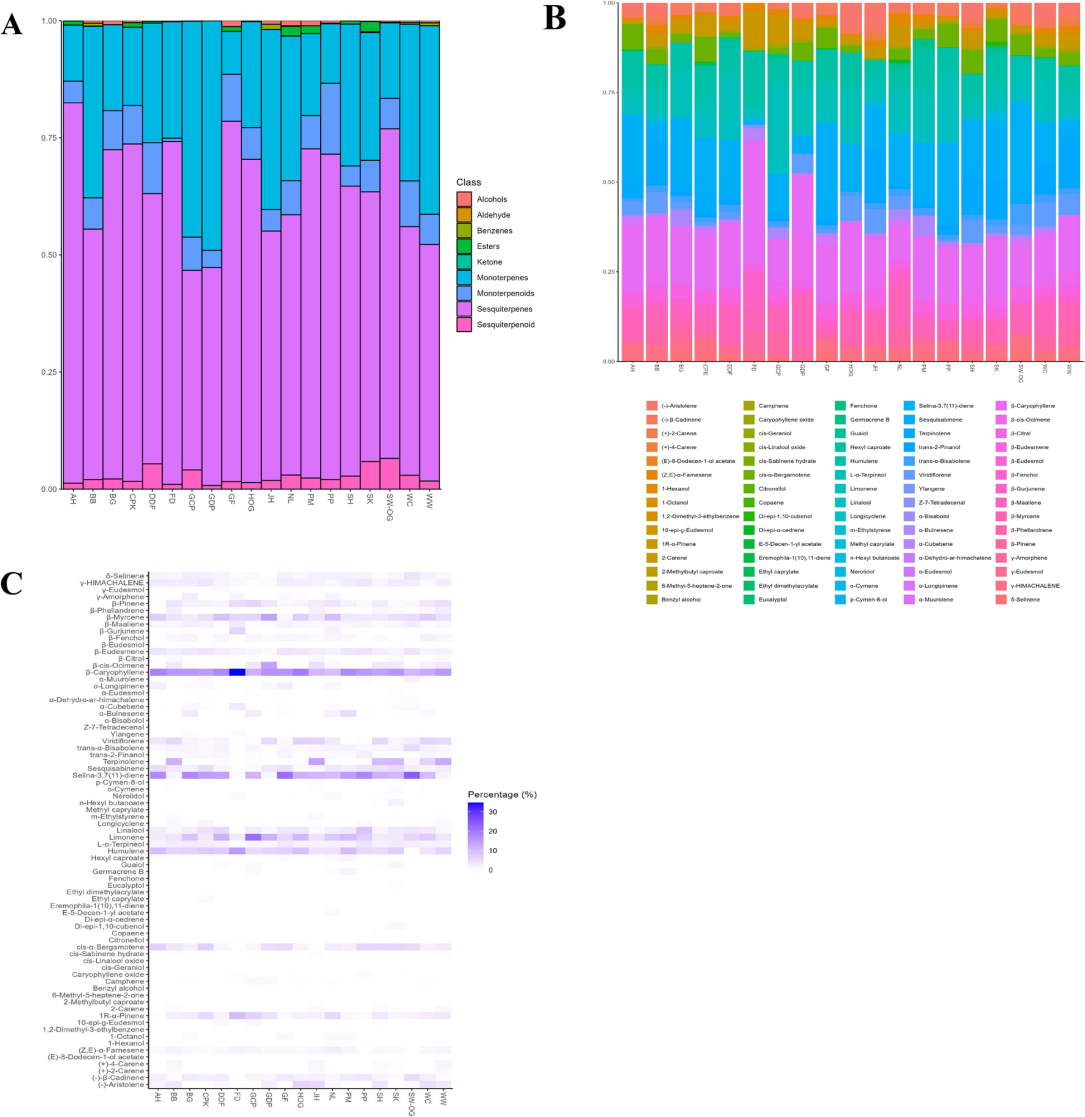
**A** Bar plot of the proportion of the 9 chemical classes (y-axis) found in all dried cannabis flower samples (x-axis). **B** Bar plot of the proportion of the 75 identifiable volatile compounds (y-axis) in each dried cannabis (x-axis). **C** Heatmap of percentage area normalization of each identifiable volatile compound in each strain

The tentative volatile compound profiles with their normalized peak areas in each sample were summarized in Table 2; Fig. 2A, showing 75 identifiable compounds divided into nine classes (alcohols, aldehyde, benzenes, esters, ketone, monoterpenes, monoterpenoids, sesquiterpenes, and sesquiterpenoids). According to Fig. 2A, three major classes found in all dried cannabis flowers were sesquiterpenes (42.60–76.92%), monoterpenes (9.15–48.99%), and monoterpenoids (0.71–15.15%), respectively. The minor classes can also be detected; sesquiterpenoids (0.78–6.57%), alcohols (0.03–1.26%), aldehyde (0.11%), benzenes (0.05–1.11%), esters (0.02–2.18%) and ketone (0.01–0.07%). Each cannabis sample provided a characteristic volatile profile leading to a distinctive aroma. The major and minor volatile compounds of each sample were summarized in a bar plot, as can be seen in Fig. 2B. β-caryophyllene and selina-3,7(11)-diene were the main compounds found in most cannabis flower samples. β-caryophyllene was the predominant sesquiterpene presenting the highest percent area normalization in FD (34.72%), HOG (19.75%), AH (18.62%), DDF (18.05%), PM (17.64%), GDP (16.27%), SK (16.16%), BB (15.91%), WW (15.51%), CPK (15.04%), SH (13.90%), and WC (12.89%). While cannabis flowers of SW-OG, GF, PP, BG, and NL had selina-3,7(11)-diene as a dominant sesquiterpene, showing the highest percentage values of 24.38%, 21.57%, 18.29%, 17.90% and 10.08%, respectively. In contrast, it showed low percentage values in cannabis cultivars of WW (1.80%), BB (1.34%), GDP (1.25%), and FD (0.58%). JH and GCP differed from all other samples. Terpinolene and limonene were the dominant monoterpenes detected in JH and GCP exhibiting the highest percentage contents of 13.82% and 21.45%, respectively. Terpinolene was also the major volatile compound in the cannabis flowers of WW (12.94%), BB (11.74%), SK (10.61%), SH (10.02%), and WC (6.75%). The cannabis samples of GDP, HOG, PM, BG, NL, WC, PP, and SW-OG had limonene as one of the major volatile compounds, presenting 11.60%, 10.51%, 9.97%, 8.84%, 8.34%,7.76%, 7.04%, and 5.97%, respectively. The other major volatile compounds can be found in most samples; for example, β-myrcene, humulene, linalool, cis-α-bergamotene, and 1R-α-pinene. The previous study (Stenerson and Halpenny 2017) developed an HS-SPME-GC-MS approach (using DVB/CAR/PDMS fiber) to characterize volatile terpenes from hemp inflorescences. They suggested that the DVB/CAR/PDMS fiber provided enough sensitivity to produce adequate MS spectra for identification purposes. The optimal HS-SPME-GC-MS condition allowed 45 identifiable compounds, with caryophyllene showing the most abundant terpene, followed by α-pinene and limonene.

**Table 2:** Tentative identification of compounds in 19 dried cannabis flower sample. Compounds are confirmed from the MS match score of >650, and *I* difference of within ±30 units from literature values

**Peak No.**	**RT (min)**	**Tentative Compound**	***I* (exp)**	***Δ I***	**Match score**	**%Average area normalization** (*n*=5) **±SD**						
						**SK OG**	**PP**	**JH**	**WC**	**BB**	**WW**	**NL**
		**Alcohols**										
16	10.32	1-Hexanol	1334	21	929	0.19 ± 0.02	0.25 ± 0.02	0.07 ± 0.01	0.10 ± 0.01	0.12 ± 0.02	0.10 ± 0.00	0.23 ± 0.01
32	15.68	1-Octanol	1537	20	876	-	-	-	-	-	-	0.83 ± 0.04
62	23.66	p-Cymen-8-ol	1820	25	921	0.06 ± 0.00	0.23 ± 0.03	0.63 ± 0.09	0.14 ± 0.01	0.39 ± 0.07	0.32 ± 0.04	-
63	24.04	Benzyl alcohol	1847	23	882	0.08 ± 0.00	0.05 ± 0.01	0.06 ± 0.02	0.16 ± 0.01	0.03 ± 0.00	0.03 ± 0.00	0.03 ± 0.00
		**Aldehyde**										
65	26.71	Z-7-Tetradecenal	1955	5	698	-	-	-	-	-	-	0.11 ± 0.01
		**Benzenes**										
18	11.53	1,2-Dimethyl-3-ethylbenzene	1370	1	768	0.01 ± 0.00	0.01 ± 0.00	0.15 ± 0.03	0.05 ± 0.01	0.12 ± 0.01	0.13 ± 0.02	-
22	12.60	m-Ethylstyrene	1411	13	841	0.08 ± 0.01	0.04 ± 0.00.	0.96 ± 0.06	0.29 ± 0.03	0.51 ± 0.06	0.50 ± 0.05	-
		**Esters**										
11	7.80	Ethyl dimethylacrylate	1211	6	832	-	0.02 ± 0.00	-	-	-	-	-
17	11.37	Methyl caprylate	1364	21	835	-	-	-	-	-	-	-
20	11.99	n-Hexyl butanoate	1401	13	870	-	-	-	-	-	-	0.05 ± 0.00
21	12.57	Ethyl caprylate	1409	26	945	-	-	-	-	-	-	-
23	13.03	2-Methylbutyl caproate	1440	8	991	-	-	-	-	-	-	-
39	17.30	Hexyl caproate	1595	7	921	-	-	-	-	-	-	1.06 ± 0.09
59	22.00	E-5-Decen-1-yl acetate	1768	5	896	-	-	-	-	-	-	0.87 ± 0.01
66	26.86	(E)-8-Dodecen-1-ol acetate	1961	4	838	-	-	-	-	-	-	0.08 ± 0.01
		**Ketone**										
15	10.06	6-Methyl-5-heptene-2-one	1323	15	866	0.03 ± 0.00	0.07 ± 0.01	0.01 ± 0.00	0.02 ± 0.00	0.02 ± 0.00	0.01 ± 0.00	0.03 ± 0.01
		**Monoterpenes**										
1	5.18	1R-α-Pinene	1017	4	899	1.91 ± 0.15	0.82 ± 0.08	2.17 ± 0.08	2.76 ± 0.16	2.68 ± 0.18	3.37 ± 0.10	5.60 ± 0.38
2	5.58	Camphene	1059	12	866	0.22 ± 0.02	0.18 ± 0.02	0.11 ± 0.01	0.45 ± 0.03	0.16 ± 0.02	0.21 ± 0.05	0.42 ± 0.08
3	6.02	β-Pinene	1104	8	878	1.08 ± 0.57	0.83 ± 0.09	2.65 ± 0.08	3.58 ± 0.19	3.87 ± 0.25	4.69 ± 0.16	4.30 ± 0.33
4	6.62	2-Carene	1138	1	846	-	-	0.79 ± 0.03	0.25 ± 0.01	0.83 ± 0.06	1.10 ± 0.05	-
5	6.71	b-Myrcene	1151	10	925	5.20 ± 0.38	3.17 ± 0.24	3.76 ± 0.36	7.79 ± 0.42	4.32 ± 0.47	4.61 ± 0.22	9.94 ± 0.27
6	6.85	(+)-4-Carene	1153	4	744	-	-	1.42 ± 0.17	0.35 ± 0.02	1.30 ± 0.14	1.56 ± 0.26	-
7	7.08	(+)-2-Carene	1168	10	836	0.03 ± 0.01	-	0.99 ± 0.14	-	0.78 ± 0.11	0.96 ± 0.20	-
8	7.33	Limonene	1193	6	916	5.97 ± 0.42	7.04 ± 0.46	4.07 ± 0.28	7.76 ± 0.39	3.92 ± 0.36	3.78 ± 0.20	8.34 ± 0.23
9	7.46	β-Phellandrene	1201	10	923	-	-	2.36 ± 0.15	1.06 ± 0.06	2.29 ± 0.18	2.68 ± 0.25	0.88 ± 0.07
12	8.24	β-cis-Ocimene	1232	3	877	0.50 ± 0.05	0.26 ± 0.02	4.7 ± 0.71	2.41 ± 0.14	3.44 ± 0.41	3.32 ± 0.16	0.96 ± 0.07
13	8.70	o-Cymene	1253	22	907	-	-	0.39 ± 0.02	0.16 ± 0.02	0.29 ± 0.05	0.27 ± 0.04	-
14	8.94	Terpinolene	1265	18	935	1.19 ± 0.09	0.40 ± 0.04	13.82 ± 1.46	6.75 ± 0.24	11.74 ± 1.18	12.92 ± 0.52	0.44 ± 0.04
45	19.53	β-Citral	1664	16	758	-	-	1.22 ± 0.29	0.15 ± 0.01	0.99 ± 0.16	0.77 ± 0.11	-
		**Monoterpenoids**										
10	7.72	Eucalyptol	1207	6	886	-	0.01 ± 0.00	-	0.29 ± 0.04	-	-	0.12 ± 0.02
19	11.70	Fenchone	1390	Not available	895	0.12 ± 0.01	0.07 ± 0.01	0.06 ± 0.01	0.12 ± 0.01	0.10 ± 0.01	0.10 ± 0.01	0.13 ± 0.00
24	13.06	cis-Linalool oxide	1441	3	865	0.05 ± 0.01	0.02 ± 0.00	-	-	-	-	-
25	13.60	cis-Sabinene hydrate	1461	2	718	-	0.03 ± 0.00	0.46 ± 0.08	0.26 ± 0.02	0.46 ± 0.05	0.44 ± 0.03	-
30	15.39	Linalool	1526	21	912	3.80 ± 0.29	8.83 ± 0.38	0.72 ± 0.06	3.47 ± 0.21	1.20 ± 0.06	1.14 ± 0.05	2.53 ± 0.46
31	15.57	trans-2-Pinanol	1533	11	811	-	2.32 ± 0.20	0.85 ± 0.11	1.36 ± 0.10	0.78 ± 0.18	0.81 ± 0.07	0.81 ± 0.05
35	16.35	b-Fenchol	1561	15	889	-	-	0.70 ± 0.05	2.21 ± 0.13	1.40 ± 0.06	1.35 ± 0.08	1.64 ± 0.07
46	19.62	L-α-Terpineol	1679	11	870	2.51 ± 0.12	3.76 ± 0.04	1.80 ± 0.29	1.92 ± 0.12	2.44 ± 0.12	2.27 ± 0.09	2.05 ± 0.06
57	21.56	Citronellol	1752	13	783	-	-	-	-	-	-	-
61	23.58	cis-Geraniol	1817	20	897	0.03 ± 0.00	0.11 ± 0.01	-	0.09 ± 0.05	0.30 ± 0.08	0.32 ± 0.03	-
		**Sesquiterpenes**										
26	13.73	α-Cubebene	1466	3	882	0.90 ± 0.06	0.35 ± 0.01	0.33 ± 0.04	0.31 ± 0.02	0.19 ± 0.01	0.17 ± 0.01	0.66 ± 0.02
27	13.98	Ylangene	1475	16	905	0.26 ± 0.03	0.24 ± 0.00	0.12 ± 0.01	0.11 ± 0.01	0.04 ± 0.01	0.04 ± 0.01	0.20 ± 0.01
28	14.59	Copaene	1497	5	856	0.06 ± 0.01	0.02 ± 0.00	-	-	-	-	0.04 ± 0.00
29	15.04	Di-epi-α-cedrene	1514	11	877	0.1 ± 0.00	0.12 ± 0.02	0.01 ± 0.00	0.03 ± 0.01	0.09 ± 0.00	0.09 ± 0.01	0.08 ± 0.08
33	15.99	α-Longipinene	1548	23	857	-	-	-	-	-	-	1.27 ± 0.14
34	16.29	Longicyclene	1558	4	869	0.30 ± 0.03	0.76 ± 0.17	0.28 ± 0.13	0.16 ± 0.06	1.10 ± 0.34	1.03 ± 0.42	-
36	16.47	cis-α-Bergamotene	1565	3	842	5.65 ± 0.16	6.24 ± 0.07	-	2.16 ± 0.14	3.81 ± 0.36	3.29 ± 1.05	3.18 ± 0.06
37	16.60	(-)-Aristolene	1570	2	858	0.81 ± 0.09	-	5.84 ± 0.53	4.42 ± 0.36	3.54 ± 0.17	3.39 ± 0.15	1.22 ± 0.06
38	16.94	b-Caryophyllene	1582	13	925	10.53 ± 0.48	15.47 ± 0.12	10.82 ± 0.57	12.89 ± 0.69	15.91 ± 0.42	15.51 ± 0.32	9.58 ± 0.11
40	17.44	β-Gurjunene	1600	6	877	-	-	-	-	-	-	2.16 ± 0.11
41	17.81	α-Bulnesene	1613	11	854	-	-	0.25 ± 0.02	1.10 ± 0.08	-	-	2.24 ± 0.15
42	18.56	Sesquisabinene	1641	7	854	2.58 ± 0.08	4.11 ± 0.13	0.98 ± 0.51	1.52 ± 0.05	3.26 ± 0.20	2.94 ± 0.15	3.71 ± 0.10
43	18.77	Humulene	1648	19	908	-	5.92 ± 0.07	4.26 ± 2.24	3.97 ± 1.90	6.61 ± 0.20	6.22 ± 0.17	4.24 ± 0.09
44	19.13	β-Maaliene	1661	10	886	2.67 ± 0.08	1.91 ± 0.08	1.33 ± 0.18	0.63 ± 0.03	1.27 ± 0.09	1.14 ± 0.08	1.44 ± 0.08
47	19.84	Viridiflorene	1687	10	853	2.66 ± 0.10	0.44 ± 0.02	6.49 ± 0.60	6.51 ± 0.22	5.97 ± 0.45	5.77 ± 0.36	3.41 ± 0.07
48	20.02	γ-HIMACHALENE	1693	18	852	3.04 ± 0.07	3.84 ± 0.13	2.07 ± 0.55	0.96 ± 0.07	3.07 ± 0.28	2.70 ± 0.12	1.11 ± 0.03
49	20.12	β-Eudesmene	1697	20	880	3.92 ± 0.11	4.27 ± 0.17	2.63 ± 0.81	1.71 ± 0.06	2.50 ± 0.11	2.27 ± 0.11	2.04 ± 0.03
50	20.30	(-)-β-Cadinene	1704	16	838	5.36 ± 1.45	3.71 ± 0.08	2.28 ± 0.37	2.40 ± 0.06	0.60 ± 0.21	0.65 ± 0.17	1.58 ± 0.02
51	20.84	δ-Selinene	1712	16	943	4.21 ± 0.12	1.75 ± 0.2	2.31 ± 0.27	2.12 ± 0.09	1.39 ± 0.20	1.21 ± 0.14	2.33 ± 0.08
52	20.87	(Z,E)-α-Farnesene	1713	12	941	-	0.26 ± 0.01	1.59 ± 0.13	1.43 ± 0.16	2.17 ± 0.20	1.81 ± 0.14	2.69 ± 0.07
53	20.97	γ-Amorphene	1729	10	936	-	-	-	-	-	-	1.52 ± 0.04
54	21.00	α-Muurolene	1730	4	847	2.22 ± 0.29	0.88 ± 0.05	0.46 ± 0.12	0.36 ± 0.02	0.29 ± 0.03	0.25 ± 0.07	-
55	21.08	Eremophila-1(10),11-diene	1734	9	820	-	-	-	-	-	-	-
58	21.64	Selina-3,7(11)-diene	1755	5	910	24.38 ± 0.90	18.29 ± 0.31	11.00 ± 0.82	10.10 ± 0.39	1.34 ± 0.69	1.80 ± 0.11	10.08 ± 0.36
60	22.85	Germacrene B	1800	19	885	-	-	-	-	-	-	0.59 ± 0.06
64	24.95	α-Dehydro-ar-himachalene	1883	1	747	0.52 ± 0.04	0.26 ± 0.01	0.07 ± 0.01	0.04 ± 0.01	0.12 ± 0.01	0.12 ± 0.01	0.15 ± 0.01
67	27.41	Caryophyllene oxide	1973	16	848	0.20 ± 0.02	0.58 ± 0.09	0.10 ± 0.06	0.19 ± 0.03	0.23 ± 0.12	0.11 ± 0.03	-
		**Sesquiterpenoids**										
56	21.38	trans-α-Bisabolene	1745	4	944	5.75 ± 0.25	1.38 ± 0.12	1.59 ± 0.17	2.32 ± 0.16	1.81 ± 0.26	1.59 ± 0.12	1.94 ± 0.09
68	28.03	Nerolidol	2010	2	899	0.23 ± 0.04	0.33 ± 0.08	0.07 ± 0.05	0.14 ± 0.04	0.09 ± 0.04	0.05 ± 0.01	0.89 ± 0.06
69	29.09	Di-epi-1,10-cubenol	2055	1	773	-	-	-	-	-	-	-
70	29.55	Guaiol	2064	30	830	0.05 ± 0.00	-	-	0.07 ± 0.02	-	-	-
71	29.92	10-epi-g-Eudesmol	2080	28	913	0.05 ± 0.01	-	-	0.08 ± 0.02	-	-	-
72	32.32	γ-Eudesmol	2188	11	864	-	-	-	-	-	-	-
73	32.55	α-Bisabolol	2198	17	883	0.32 ± 0.06	0.22 ± 0.06	0.14 ± 0.08	0.24 ± 0.07	0.11 ± 0.05	0.06 ± 0.01	0.17 ± 0.01
74	32.66	α-Eudesmol	2203	26	873	0.08 ± 0.11	0.04 ± 0.01	0.02 ± 0.01	0.04 ± 0.01	0.01 ± 0.00	0.01 ± 0.00	0.01 ± 0.00
75	32.84	β-Eudesmol	2211	28	836	0.08 ± 0.02	0.08 ± 0.01	0.05 ± 0.02	0.07 ± 0.01	0.03 ± 0.00	0.02 ± 0.00	0.01 ± 0.00
		**SUM**				100	100	100	100	100	100	100
		**Alcohols**				0.33	0.53	0.76	0.40	0.54	0.45	1.09
		**Aldehyde**				0.00	0.00	0.00	0.00	0.00	0.00	0.11
		**Benzenes**				0.09	0.05	1.11	0.35	0.63	0.63	0.00
		**Esters**				0.00	0.02	0.00	0.00	0.00	0.00	2.06
		**Ketone**				0.03	0.07	0.01	0.02	0.02	0.01	0.03
		**Monoterpenes**				16.11	12.70	38.44	33.46	36.63	40.23	30.87
		**Monoterpenoids**				6.52	15.15	4.58	9.73	6.67	6.45	7.29
		**Sesquiterpenes**				70.35	69.43	53.23	53.10	53.47	50.51	55.55
		**Sesquiterpenoid**				6.57	2.06	1.86	2.95	2.05	1.73	3.01

**Peak No.**	**%Average area normalization** (*n*=5) **± SD**											
	**GDP**	**GCP**	**FD**	**DDF**	**CPK**	**PM**	**SK**	**BG**	**GF**	**AH**	**SH**	**HOG**
16	-	0.04 ± 0.00	0.02 ± 0.01	0.09 ± 0.01	0.21 ± 0.01	0.19 ± 0.01	0.19 ± 0.04	0.06 ± 0.00	0.03 ± 0.00	0.07 ± 0.00	0.05 ± 0.00	0.03 ± 0.00
32	-	0.01 ± 0.01	-	-	-	0.88 ± 0.05	-	0.79 ± 0.04	1.22 ± 0.74	-	-	-
62	-	-	-	0.02 ± 0.01	0.16 ± 0.17	-	-	-	-	-	-	-
63	0.05 ± 0.00	0.04 ± 0.00	0.13 ± 0.01	0.05 ± 0.01	0.06 ± 0.00	0.01 ± 0.00	-	-	-	-	-	-
65	-	-	-	-	-	-	-	-	-	-	-	-
18	-	-	-	-	-	-	-	-	-	-	-	-
22	-	-	-	-	-	-	-	-	-	-	-	-
11	-	-	-	0.36 ± 0.03	0.02 ± 0.01	-	-	-	-	-	-	-
17	-	-	-	-	0.04 ± 0.00	-	-	-	-	-	-	-
20	-	0.02 ± 0.00	0.04 ± 0.00	-	-	0.07 ± 0.01	2.18 ± 0.08	-	0.07 ± 0.01	0.04 ± 0.01	0.24 ± 0.05	0.01 ± 0.00
21	-	-	-	-	0.91 ± 0.02	-	-	-	-	-	-	-
23	-	-	-	-	-	-	-	-	0.13 ± 0.02	0.06 ± 0.02	0.31 ± 0.02	-
39	-	-	-	-	-	1.60 ± 0.08	-	-	0.75 ± 0.59	0.43 ± 0.15	0.12 ± 0.04	0.16 ± 0.01
59	-	-	-	-	-	-	-	-	0.08 ± 0.01	0.34 ± 0.11	-	-
66	-	-	-	-	-	-	-	-	-	-	-	-
15	-	0.01 ± 0.00	0.03 ± 0.01	0.02 ± 0.01	0.02 ± 0.00	-	0.15 ± 0.02	0.02 ± 0.00	0.01 ± 0.00	0.01 ± 0.00	0.02 ± 0.00	0.01 ± 0.00
1	4.50 ± 0.17	6.76 ± 0.21	9.97 ± 0.18	1.76 ± 0.21	5.65 ± 0.46	1.22 ± 0.07	1.35 ± 0.07	2.95 ± 0.27	0.71 ± 0.06	0.42 ± 0.03	3.46 ± 0.36	2.02 ± 0.13
2	0.78 ± 0.07	1.01 ± 0.18	0.27 ± 0.07	0.36 ± 0.04	0.17 ± 0.02	0.36 ± 0.05	0.06 ± 0.01	0.38 ± 0.07	0.16 ± 0.02	0.07 ± 0.01	0.20 ± 0.05	0.45 ± 0.05
3	3.68 ± 0.17	6.33 ± 0.21	5.28 ± 0.14	1.52 ± 0.15	1.99 ± 0.19	2.12 ± 0.12	0.56 ± 0.03	2.19 ± 0.27	0.71 ± 0.07	0.47 ± 0.05	3.14 ± 0.29	1.70 ± 0.15
4	-	-	-	-	-	-	0.33 ± 0.03	-	-	-	0.59 ± 0.05	-
5	13.98 ± 0.32	6.50 ± 0.32	6.08 ± 0.48	9.48 ± 0.83	4.81 ± 0.50	3.50 ± 0.16	4.61 ± 0.34	2.93 ± 0.41	1.44 ± 0.16	7.74 ± 0.65	3.24 ± 0.42	7.33 ± 0.41
6	-	-	-	-	-	-	0.5 ± 0.15	-	-	-	0.83 ± 0.06	-
7	-	-	-	-	-	-	0.33 ± 0.12	-	-	-	0.64 ± 0.03	-
8	11.6 ± 0.34	21.45 ± 0.94	3.14 ± 0.24	11.58 ± 1.00	3.40 ± 0.34	9.97 ± 0.32	3.17 ± 0.23	8.84 ± 0.87	5.86 ± 0.44	2.93 ± 0.28	2.95 ± 0.29	10.51 ± 0.38
9	-	-	-	-	-	-	0.83 ± 0.19	-	-	-	1.42 ± 0.10	-
12	14.18 ± 0.14	3.60 ± 0.22	0.02 ± 0.01	0.13 ± 0.01	0.12 ± 0.01	0.04 ± 0.00	3.29 ± 0.24	0.59 ± 0.09	0.07 ± 0.01	0.12 ± 0.02	3.48 ± 0.21	0.09 ± 0.01
13	-	-	-	0.02 ± 0.00	0.04 ± 0.03	-	0.61 ± 0.04	-	-	-	0.27 ± 0.01	0.03 ± 0.00
14	0.28 ± 0.02	0.46 ± 0.10	0.08 ± 0.01	0.64 ± 0.04	0.51 ± 0.06	0.28 ± 0.03	10.61 ± 0.99	0.48 ± 0.06	0.19 ± 0.02	0.21 ± 0.09	10.02 ± 0.37	0.48 ± 0.02
45	-	-	-	-	-	-	1.06 ± 0.16	-	-	-	-	-
10	-	-	-	-	-	-	0.39 ± 0.17	-	-	-	-	-
19	0.11 ± 0.02	0.24 ± 0.01	-	0.04 ± 0.00	0.13 ± 0.01	0.07 ± 0.01	0.10 ± 0.02	0.11 ± 0.00	0.18 ± 0.01	0.05 ± 0.01	0.02 ± 0.00	0.02 ± 0.00
24	-	-	-	0.01 ± 0.00	-	-	-	-	-	-	-	-
25	-	-	-	0.03 ± 0.00	0.02 ± 0.02	-	-	-	-	-	-	-
30	1.22 ± 0.07	2.96 ± 0.18	0.21 ± 0.02	5.75 ± 0.55	4.38 ± 0.18	3.43 ± 0.15	3.06 ± 0.04	2.35 ± 0.14	3.54 ± 0.09	2.78 ± 0.15	1.44 ± 0.15	3.69 ± 0.14
31	-	0.66 ± 0.24	0.03 ± 0.01	1.19 ± 0.07	1.08 ± 0.19	0.59 ± 0.06	-	1.03 ± 0.05	1.52 ± 0.33	-	-	-
35	1.06 ± 0.04	1.41 ± 0.08	0.25 ± 0.16	1.61 ± 0.20	-	1.21 ± 0.10	-	1.92 ± 0.08	1.59 ± 0.13	-	0.68 ± 0.04	1.17 ± 0.04
46	1.28 ± 0.04	1.80 ± 0.06	0.16 ± 0.05	2.24 ± 0.12	2.51 ± 0.15	1.84 ± 0.09	3.17 ± 0.16	2.92 ± 0.13	3.20 ± 0.11	1.80 ± 0.40	2.19 ± 0.19	1.91 ± 0.09
57	-	-	0.06 ± 0.01	-	-	-	-	-	-	-	-	-
61	-	-	-	0.01 ± 0.00	0.09 ± 0.04	-	-	-	-	-	-	-
26	-	0.09 ± 0.01	3.17 ± 0.04	0.24 ± 0.02	0.48 ± 0.02	0.16 ± 0.01	0.53 ± 0.04	1.20 ± 0.02	0.31 ± 0.01	0.22 ± 0.01	0.14 ± 0.01	0.17 ± 0.00
27	-	0.06 ± 0.01	0.88 ± 0.05	0.18 ± 0.03	0.14 ± 0.02	0.10 ± 0.01	0.14 ± 0.02	0.53 ± 0.03	0.24 ± 0.01	0.17 ± 0.01	0.11 ± 0.00	0.15 ± 0.00
28	0.07 ± 0.00	0.05 ± 0.00	-	-	-	0.06 ± 0.01	-	-	0.19 ± 0.01	0.19 ± 0.02	0.14 ± 0.01	0.03 ± 0.01
29	-	-	-	0.02 ± 0.00	0.12 ± 0.01	-	-	0.03 ± 0.00	-	-	-	-
33	0.99 ± 0.14	-	-	-	0.92 ± 0.12	-	-	0.53 ± 0.16	2.43 ± 0.11	2.36 ± 0.13	-	-
34	-	-	-	0.04 ± 0.00	1.36 ± 0.23	-	-	-	-	-	-	-
36	4.90 ± 0.12	1.68 ± 0.17	0.14 ± 0.07	1.31 ± 0.06	6.94 ± 0.20	2.55 ± 0.10	6.26 ± 0.68	2.32 ± 0.10	5.63 ± 0.06	7.00± 0.15	6.41 ± 0.10	1.84 ± 0.08
37	2.72 ± 0.11	-	-	0.95 ± 0.11	-	-	-	-	-	1.76 ± 0.13	3.96 ± 0.28	6.41 ± 0.11
38	16.27 ± 0.17	12.02 ± 0.13	34.72 ± 0.25	18.05 ± 0.4	15.04 ± 0.12	17.64 ± 0.31	16.16 ± 0.57	15.7 ± 0.45	16.29 ± 0.21	18.62 ± 0.28	13.90 ± 0.58	19.75 ± 0.19
40	-	0.16 ± 0.06	6.37 ± 0.15	-	-	-	-	-	-	-	-	-
41	-	2.98 ± 0.30	0.36 ± 0.06	-	-	5.39 ± 0.22	-	3.03 ± 0.09	0.47 ± 0.03	0.22 ± 0.04	0.19 ± 0.01	0.05 ± 0.02
42	3.43 ± 0.06	0.89 ± 0.02	0.41 ± 0.04	1.46 ± 0.09	5.37 ± 0.26	2.62 ± 0.05	5.62 ± 0.35	2.15 ± 0.06	4.90 ± 0.11	5.23 ± 0.35	4.42 ± 0.12	0.83 ± 0.07
43	6.61 ± 0.04	5.28 ± 0.19	14.94 ± 0.31	7.62 ± 0.16	8.20 ± 0.30	10.01 ± 0.11	7.07 ± 0.55	6.74 ± 0.18	6.94 ± 0.10	9.49 ± 0.20	5.53 ± 0.19	9.23 ± 0.13
44	0.62 ± 0.06	0.49 ± 0.15	3.26 ± 0.25	0.66 ± 0.05	2.23 ± 0.93	1.64 ± 0.13	1.37 ± 0.15	2.49 ± 0.05	1.98 ± 0.07	1.67 ± 0.30	1.07 ± 0.22	1.31 ± 0.08
47	5.44 ± 0.14	0.39 ± 0.06	-	1.91 ± 0.22	0.99 ± 0.10	-	1.80 ± 0.08	1.57 ± 0.03	0.80 ± 0.04	3.75 ± 0.28	6.12 ± 0.29	6.85 ± 0.20
48	0.60 ± 0.09	0.46 ± 0.04	1.25 ± 0.06	1.48 ± 0.05	3.99 ± 0.22	2.97 ± 0.09	3.17 ± 0.23	2.62 ± 0.07	3.09 ± 0.07	3.13 ± 0.16	1.68 ± 0.04	2.51 ± 0.06
49	0.84 ± 0.14	1.45 ± 0.07	2.19 ± 0.11	2.80 ± 0.13	4.12 ± 0.24	3.77 ± 0.12	1.65 ± 0.04	3.52 ± 0.10	3.93 ± 0.09	3.79 ± 0.19	2.67 ± 0.05	3.54 ± 0.07
50	1.61 ± 0.26	1.73 ± 0.09	-	2.81 ± 0.25	2.64 ± 0.16	3.18 ± 0.09	0.60 ± 0.04	3.36 ± 0.08	3.44 ± 0.08	2.42 ± 0.19	1.54 ± 0.04	2.25 ± 0.10
51	-	0.99 ± 0.04	0.61 ± 0.04	1.80 ± 0.29	2.50 ± 0.16	2.01 ± 0.12	1.27 ± 0.08	2.64 ± 0.10	2.41 ± 0.12	1.8 ± 0.39	0.88 ± 0.01	1.14 ± 0.06
52	1.05 ± 0.03	1.74 ± 0.12	2.26 ± 0.15	1.06 ± 0.12	0.65 ± 0.10	1.51 ± 0.08	0.86 ± 0.04	1.01 ± 0.02	1.07 ± 0.03	0.90 ± 0.20	0.95 ± 0.05	0.70 ± 0.04
53	-	-	1.51 ± 0.06	-	-	-	0.15 ± 0.02	2.75 ± 0.58	0.92 ± 0.02	0.45 ± 0.21	0.36 ± 0.01	0.39 ± 0.03
54	-	-	-	0.48 ± 0.05	0.94 ± 0.06	-	-	-	-	-	-	-
55	-	-	0.12 ± 0.00	-	-	-	-	-	-	-	-	-
58	1.25 ± 0.03	11.27 ± 0.27	0.58 ± 0.04	14.55 ± 1.18	14.91 ± 0.62	14.73 ± 0.30	10.70 ± 0.47	17.90 ± 0.58	21.57 ± 0.47	17.81 ± 0.69	11.70 ± 0.20	11.75 ± 0.22
60	-	0.77 ± 0.11	-	-	-	1.82 ± 0.10	-	-	0.14 ± 0.00	0.06 ± 0.00	-	-
64	-	-	-	0.13 ± 0.01	0.45 ± 0.03	-	-	-	-	-	-	-
67	0.10 ± 0.04	0.09 ± 0.02	0.43 ± 0.07	0.13 ± 0.06	0.03 ± 0.01	0.11 ± 0.05	0.24 ± 0.03	0.18 ± 0.02	0.18 ± 0.03	0.14 ± 0.03	0.11 ± 0.02	0.11 ± 0.02
56	-	1.40 ± 0.12	-	2.13 ± 0.17	1.38 ± 0.10	2.12 ± 0.20	1.79 ± 0.11	1.79 ± 0.10	1.28 ± 0.03	0.94 ± 0.21	1.64 ± 0.06	1.17 ± 0.05
68	-	0.03 ± 0.01	0.73 ± 0.19	0.27 ± 0.05	0.19 ± 0.05	0.08 ± 0.02	0.43 ± 0.03	0.10 ± 0.01	0.24 ± 0.07	0.19 ± 0.01	0.11 ± 0.03	0.10 ± 0.03
69	-	-	-	-	-	-	1.16 ± 0.09	-	-	-	-	-
70	0.23 ± 0.09	0.81 ± 0.27	0.08 ± 0.02	0.88 ± 0.18	-	-	1.32 ± 0.12	0.03 ± 0.00	0.01 ± 0.00	0.04 ± 0.01	0.37 ± 0.09	0.01 ± 0.00
71	0.32 ± 0.09	1.10 ± 0.23	0.15 ± 0.02	0.99 ± 0.15	-	-	-	0.03 ± 0.00	0.01 ± 0.00	0.04 ± 0.01	0.48 ± 0.07	0.02 ± 0.00
72	0.09 ± 0.04	0.34 ± 0.11	0.01 ± 0.01	0.54 ± 0.18	0.04 ± 0.01	-	-	-	-	-	-	-
73	-	-	-	-	-	0.15 ± 0.06	0.32 ± 0.04	0.20 ± 0.01	0.06 ± 0.03	0.07 ± 0.01	0.19 ± 0.08	0.10 ± 0.03
74	0.09 ± 0.02	0.24 ± 0.07	0.02 ± 0.01	0.26 ± 0.09	0.01 ± 0.00	-	0.42 ± 0.04	-	-	-	-	-
75	0.05 ± 0.02	0.19 ± 0.06	0.02 ± 0.01	0.32 ± 0.07	0.02 ± 0.01	-	0.43 ± 0.05	-	-	-	-	-
	100	100	100	100	100	100	100	100	100	100	100	100
	0.05	0.08	0.15	0.16	0.43	1.08	0.19	0.85	1.26	0.07	0.05	0.03
	0.00	0.00	0.00	0.00	0.00	0.00	0.00	0.00	0.00	0.00	0.00	0.00
	0.00	0.00	0.00	0.00	0.00	0.00	0.00	0.00	0.00	0.00	0.00	0.00
	0.00	0.02	0.04	0.36	0.97	1.68	2.18	0.00	1.03	0.87	0.67	0.16
	0.00	0.01	0.03	0.02	0.02	0.00	0.15	0.02	0.01	0.01	0.02	0.01
	48.99	46.11	24.85	25.49	16.70	17.49	27.30	18.35	9.15	11.95	30.25	22.61
	3.67	7.08	0.71	10.89	8.21	7.13	6.72	8.34	10.03	4.62	4.32	6.80
	46.51	42.60	73.19	57.68	72.02	70.27	57.59	70.28	76.92	81.20	61.90	68.99
	0.78	4.11	1.02	5.40	1.65	2.36	5.87	2.16	1.60	1.27	2.79	1.39

A heatmap in Fig. 2C was also generated to show the overall distribution of 75 identifiable compounds in 19 different dried cannabis flowers. The two sesquiterpenes of β-caryophyllene and selina-3,7(11)-diene showed the most significant percentages, which were in agreement with that reported in previous works (Kwaśnica et al. 2023; Cicaloni et al. 2022). The authentic aroma of β-Caryophyllene is black pepper (Sommano et al. 2020; Thurman 2020), while selina-3,7(11)-diene does not have an odor description. β-Caryophyllene, β-myrcene, limonene, and linalool can also be detected in the form of cannabis oil, showing variable amounts in each cannabis oil sample owing to diverse factors; for example, cannabis variety, environmental and cultivation conditions, storage and drying of raw plants, extraction process, and finally storage of the oil formulation (Fernández et al. 2023). Limonene is also found in various citrus plants (lemons, limes, and oranges). This compound shows a strong citrus odor. Therefore, many cosmetic and cleaning solutions use as fragrance ingredients (Meschler and Howlett 1999; Maayah et al. 2020; Thurman 2020). Myrcene shows the characteristic odor of a musky or hop-like fragrance (Hanuš and Hod 2020). Linalool exhibits an authentic floral scent. It can be found in many flowers and spices. Based on its floral scent, various commercial applications use it as an additive fragrance in hygiene products (Sommano et al. 2020; Thurman 2020). The database of volatile profiles is important in various applications. For example, it can be used to identify single cannabis strains, determine their quality, and assist breeders in developing new cannabis cultivars or aromas.

## Multivariate statistical analyses

### Hierarchical cluster analysis (HCA) and principal component analysis (PCA) for clustering

Before future research and applications, clustering of cannabis is a fundamental requirement to provide an overview of the classes and potential chemotypes in each class (Jin et al. 2021). Previous studies(Jin et al. 2021; Hazekamp, Tejkalová, and Papadimitriou 2016; Fischedick 2017) have described that THC and CBD concentrations appear to have no differentiation value. In contrast, terpene and terpenoid compositions played an important role in cannabis classification. In this study, HCA and PCA were used to identify clusters of samples. The normalized peak areas data of 75 identifiable compounds (variables) from the entire 95 samples (scores = 19 samples × 5 replications) were used to generate HCA and PCA, respectively.

**Fig. 3:**
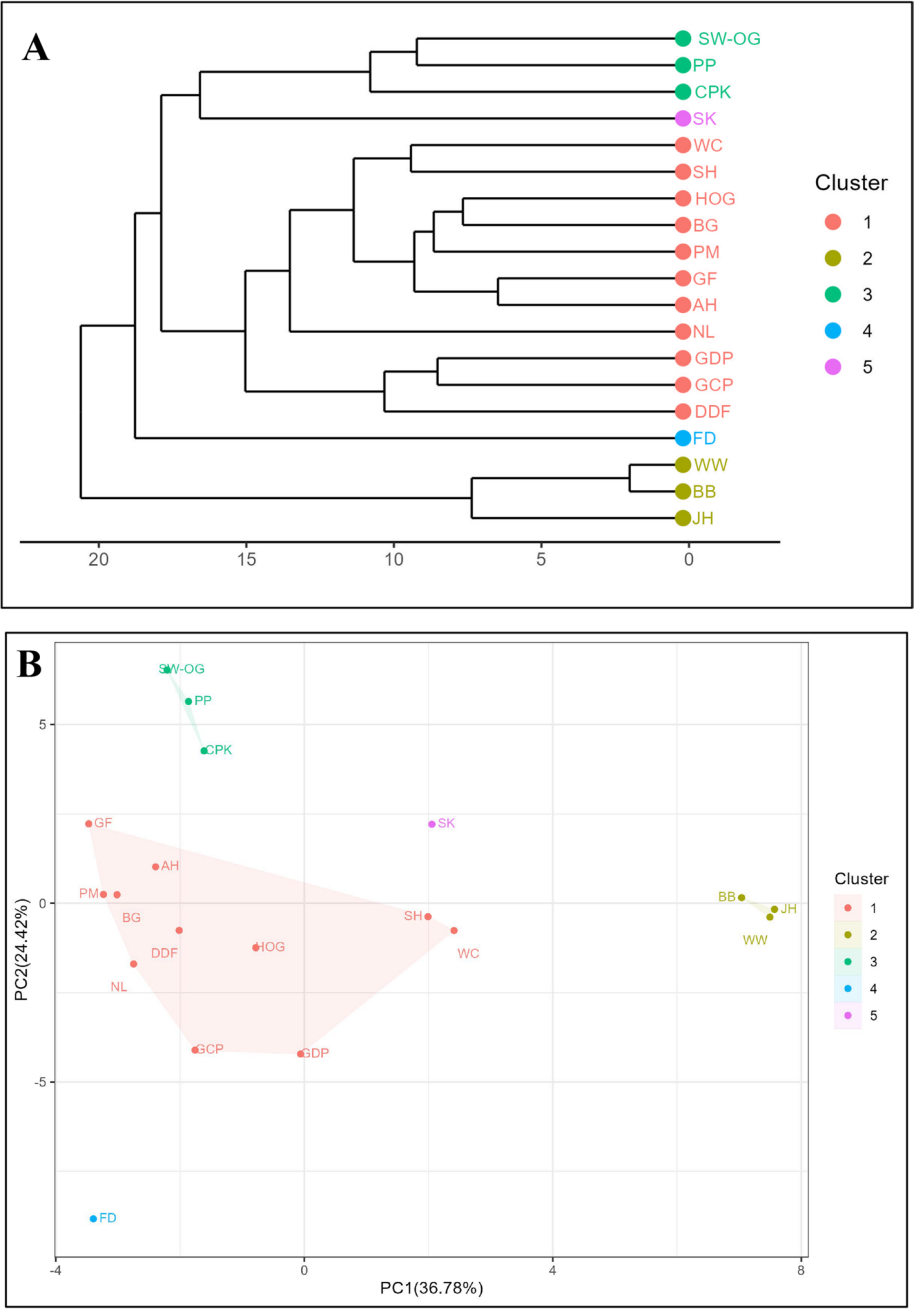
**A** Dendrogram of HCA of 19 dried cannabis flowers, presenting five main clusters. **B** PCA score plot of 19 dried cannabis flowers according to their volatiles data

The HCA result was generated as a dendrogram based on their Euclidean distances, the basis of distance between different data points, as expressed in Fig. 3A. Y- and x-axis presented a plot of the distance and the samples, respectively. A total of 19 dried cannabis flowers were obviously separated into five distinct groups, indicating that similarities and differences exist in the chemical composition of these 19 dried cannabis strains. Group I is the largest group, consisting of WC, SH, HOG, BG, PM, GF, AH, NL, GDP, GCP, and DDF. Group II is composed of WW, BB and JH. Group III includes SW-OG, PP and CPK. Groups IV and V consist solely of FD and SK, respectively.

PCA was generated in order to describe the main differences between samples based on their volatile data sets (Zheng et al. 2014). The results were shown in Fig. 3B **(**PCA score plot). Two significant principal components (PC1: x-axis and PC2: y-axis) accounting for 61.20% (PC1: 36.78% and PC2: 24.42%) of the total variance in the data matrix were used for visualization. PCA result showed five main clusters which were in good agreement with the relationship of each cannabis sample obtained from HCA (Fig. 3A), affirming the reliability of the evaluation. GC-MS chromatograms (TIC) of a representative cannabis flower sample from each cluster are provided in Figure S1 (Supplementary material). An overview of trends in key chemotypes (variables) that have the greatest influence on the class separation of the different samples (scores) was performed in the PCA biplot (with the sample overlaid on the plot) as described in Figure S2 (Supplementary material). Various main chemotypes are found in each cluster. Some key chemotypes that correlate with each cluster, along with their odor descriptions, are summarized in Table 3. However, the PCA biplot shows solely the trends of chemotypes in each cluster. To find out the most potential markers responsible for such strain, a PLS-DA approach was additionally applied next to PCA. The applications of clustering results based on their key chemotypes were discussed. For example, limonene and β-pinene are key chemotype in cluster I. In terms of pharmacological effects, limonene plays an important role in the anxiolytic, anti-stress and sedative effects of CBD by increasing serotonin and dopamine in the prefrontal cortex and hippocampus through the 5-HT1A receptor. In addition, limonene has been shown to induce apoptosis in human breast cancer cells, and this effect has been postulated to potentiate the antitumor activity of CBD in advanced stages of breast cancer (Weston-Green et al. 2021). β-Pinene exhibits the pharmacological effects as an anti-depressant and anxiolytic (Weston-Green et al. 2021). It can be implied that cannabis cultivars grouped in cluster I may exhibit these pharmacological effects, which is an interesting hypothesis to deeply study these properties in the future. Similarly, β-caryophyllene exhibits dominant properties in treating anxiety and depression. A previous study (Bahi et al. 2014) described the mechanism between β-caryophyllene and CB2 receptors-dependent manner in mice. These receptors play an important role in anxiety and stress-related disorders. β-Caryophyllene is the targeting CB2 receptors, potentially contributing to anxiolytic and anti-depressant effects. Based on the clustering results, β-Caryophyllene is a key chemotype in both cluster I and IV, indicating that cannabis cultivars in these two clusters may be effective in treating anxiety and depression.

**Table 3:** Some key chemotypes that correlate with each cluster, along with their odor descriptions

Cluster	Cultivar	Key chemotype	Odor description^a^
I	WC, SH, HOG, BG, PM, GF, AH, NL, GDP, GCP, and DDF	Limonene	Pine, herbal, and peppery
		γ-Amorphene	-
		γ-Eudesmol	Waxy, and sweet
		β-Fenchol	-
		β-Pinene	Cooling, woody, piney, and turpentine-like with a fresh minty
II	WW, BB, and JH	Terpinolene	Fresh, woody, sweet, pine, and citrus
		β-phellandrene	Mint, and terpentine
		Cis-Geraniol	Sweet, floral, fruity, rose, waxy, and citrus
		p-cymen-8-ol	Sweet, fruity, cherry, coumarin, floral, camphoreous, and cooling
		o-cymene	-
III	SW-OG, PP, and CPK	Selina-3,7(11)-diene	-
		Linalool	Citrus, orange, floral, terpy, waxy, and rose
		L-α-Terpineol	pine terpene lilac citrus woody, and floral
		cis-Linalool oxide	Earthy, floral, sweet, and woody
		δ-Selinene	-
IV	FD	Humulene	Woody, Oceanic-watery, and spicy-clove
		10-epi-γ-Eudesmol	Sweet, woody, and floral
		Eremophila-1(10),11-diene	-
		β-Caryophyllene	Sweet, woody, spice, clove and dry
		β-Gurjunene	-
V	SK	Eucalyptol	minty
		β-Eudesmol	Woody, and green
		Di-epi-1,10-cubenol	-
		n-Hexyl butanoate	-
		α-Eudesmol	-

^a^Odor description obtained from http://www.thegoodscentscompany.com

Based on commercial data on THC levels, cluster I, II, III, IV, and V contain THC levels within the ranges of 18–29%, 15–21%, 13–20%, 15%, and 13%, respectively. Their THC levels fall within a similar range suggesting that clustering based solely on cannabinoid content may not be sufficient for classification. This result is corresponding with previous studies (Jin et al. 2021; Fischedick 2017; Hazekamp, Tejkalová, and Papadimitriou 2016) which indicate that THC and CBD concentrations appear to have no differentiation value. However, terpene profiles are useful for grouping cannabis cultivars that have similar cannabinoid content (Fischedick 2017).

Regarding the source of origin, it was noticed that those cultivars labeled as sativa, indica, and hybrid overlap in the same cluster; for example, shown in cluster I, II, and III. This indicates that clustering is irrelevant to the source of origin which is in agreement with a previous study (Elzinga et al. 2015). Therefore, it is impossible to track back to their genetics owing to mixed cross-breeding several times. It has been known that most commercially available cannabis plants are hybrid (cross-breed) of sativa and indica ancestors. Thus, classification by genetics might not be effective in clustering cannabis cultivars in recent years. Consequently, a new classification by volatile chemotypes could be a reliable alternative approach, enabling the creation of well-defined and reproducible chemical profile (Hazekamp, Tejkalová, and Papadimitriou 2016). In addition, clustering based on fragrant terpenes could serve as a new determinant for users in the future. Based on commercial data, there is still a lack of comprehensive cluster analysis of cannabis strains. Therefore, these results can fulfill the need for commercial data on cluster analysis.

### Partial least squares discriminant analysis (PLS-DA) for identification of potential discriminant marker

**Fig. 4:**
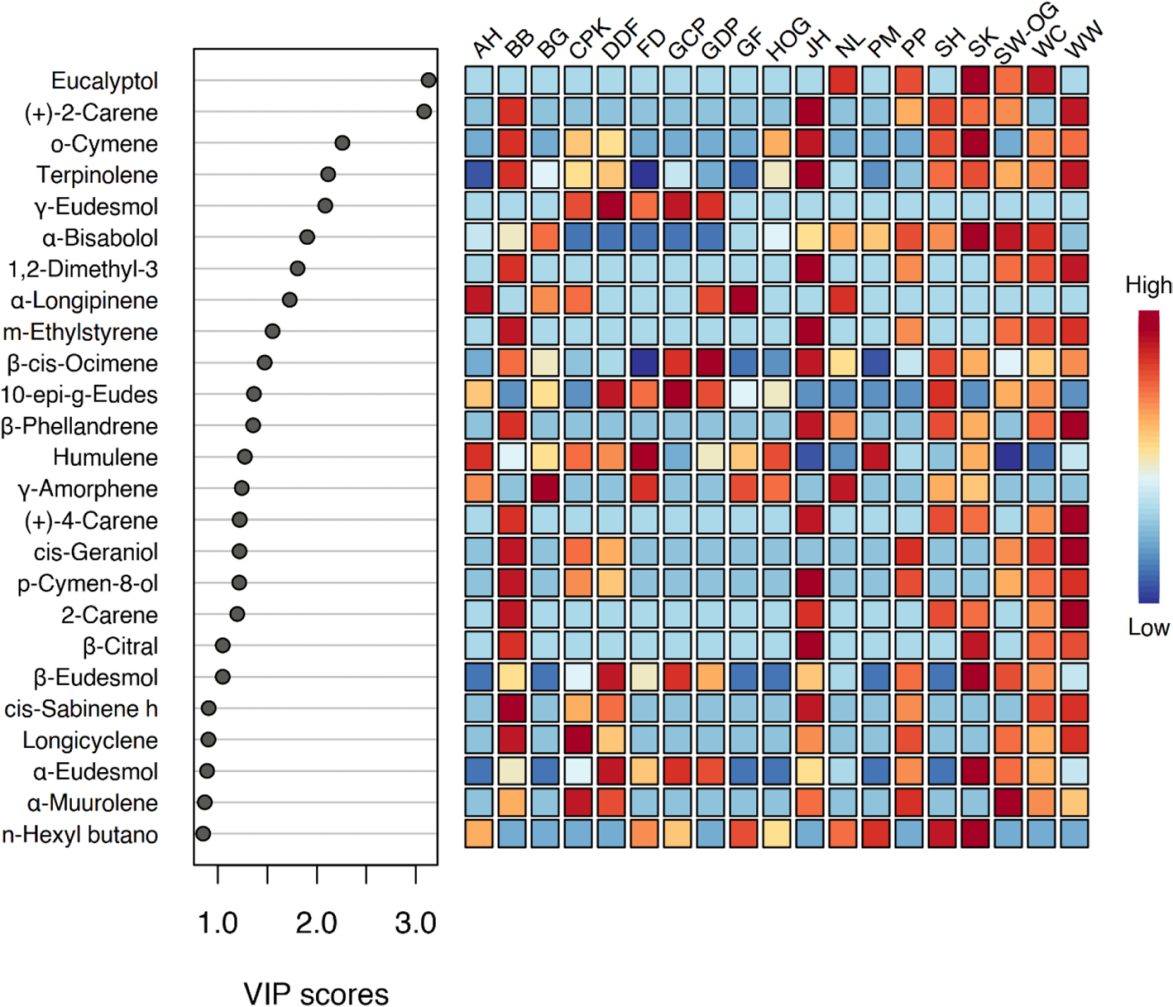
PLS-DA and VIP scores of the top twenty important volatile compounds evaluated by PLS-DA

A supervised PLS-DA is popularly used to discriminate the samples verified through Variable Importance in Projection values (VIP). The influence intensity of each variable factor on the classification and discrimination of each group of samples can be evaluated by VIP score (Feng et al. 2022). Generally, a compound or variable that shows a VIP score > 1 is regarded as significantly discriminant. In contrast, a compound with a VIP score < 0.5 is considered as unimportant variable for the model classification and discrimination (Chong and Jun 2005; Deng et al. 2021). Thus, specific volatile markers can be identified using PLS-DA (Cicaloni et al. 2022; Zheng et al. 2014). In this study, the top 20 volatile metabolites were identified by setting a threshold value of 1 for the VIP score in the PLS-DA (Deng et al. 2021) as shown in Fig. 4. Twenty volatile metabolites; including eucalyptol, (+)−2-carene, o-cymene, terpinolene, γ-eudesmol, α-bisabolol, 1,2-dimethyl-3-ethylbenzene, α-longipinene, m-ethylstyrene, β-cis-ocimene, 10-epi-γ-eudesmol, β-phellandrene, humulene, γ-amorphene, (+)−4-carene, cis-geraniol, p-cymen-8-ol, 2-carene, β-citral and β-eudesmol can be used as chemical markers to differentiate cannabis flower samples.

Compounds within the red and orange zones are characterized by high levels. Each sample can contain more than one chemical marker (Yudthavorasit et al. 2014). Eucalyptol, α-bisabolol, o-cymene and β-eudesmol perform the highest level (red) in SK compared to the other cannabis flower samples. Thus, these four compounds can be used as discriminant markers in SK. Compounds, consisting of p-cymen-8-ol, terpinolene, (+)−2-carene, m-ethylstyrene, β-citral, 1,2-dimethyl-3-ethylbenzene, are the highest level (red) in JH and these compounds can be used to identify JH from the other cannabis flower samples. Chemical markers of BB and WW are similar; however, their VIP scores are slightly different. Three major compounds of cis-geraniol, 2-carene and β-phellandrene show distinctive levels in BB and WW sample compared to the other samples. Therefore, these three compounds can be selectively used for identification of BB and WW. β-cis-Ocimene is characterized by high level in GDP and this compound can be used to distinguish GDP samples from the other cannabis flower samples. Other compounds can be used as chemical marker in GDP are γ-eudesmol, α-longipinene and 10-epi-γ-eudesmol. Compound, α-longipinene has a relatively higher VIP score in AH and GF samples than other samples. Therefore, this compound can be selectively used for identification of AH and GF samples. The difference between AH and GF are humulene and γ-amorphene. Thus, these two compounds can be used to distinguish AH and GF. γ-Eudesmol and 10-epi-γ-eudesmol show higher VIP score in DDF and GCP than the other samples. Thus, these two compounds can be used as marker compounds to differentiate DDF and GCP from the other samples. Humulene shows a high level in DDF. However, it displays low level in GCP. Thus, humulene can be used as chemical marker to separate DDF from GCP.

γ-Amorphene shows a higher level in BG and NL than in other samples. Thus, γ-amorphene can be used as marker compound, especially for BG and NL samples. However, eucalyptol is also used as chemical markers in NL; while, it shows an opposite side in BG. Therefore, eucalyptol can be used as an important compound to distinguish BG and NL. Humulene shows predominant compound in FD and PM compared to the other samples. Therefore, this compound can be used to identify FD and PM. γ-Eudesmol, 10-epi-γ-eudesmol and γ-amorphene are also play as key markers in FD, showing an opposite trend with PM sample. Eucalyptol, α-bisabolol, cis-geraniol and p-cymen-8-ol are characterized by high level (in orange) in PP and WC sample. Hence, these four compounds can be used as chemical markers in PP and WC sample. Compounds, 2-carene and β-citral can be used to differentiate PP and WW because these two compounds show an opposite trend. The main chemical markers in CPK consist of γ-eudesmol, α-longipinene, humulene and cis-geraniol (all in orange). Humulene and γ-amorphene are dominant in HOG and can be used as discriminant markers in HOG sample. Compounds, 10-epi-γ-eudesmol, β-cis-ocimene and β-phellandrene are within the orange zone which can be used as chemical markers in SH sample. α-Bisabolol (towards red zone) is one of the discriminant markers in SW-OG sample. The other marker compounds as expressed in orange in SW-OG are β-eudesmol, eucalyptol and 1,2-dimethyl-3-ethylbenzene. VIP scores of all 75 identifiable compounds were summarized in Table S3 (Supplementary material).

A previous study (Cicaloni et al. 2022) purposed ten metabolites in five different *C. sativa* female inflorescences including V1 CBD, Banana Hybrid, Green Poison, Candy BUD and Gorilla CBD. The top ten metabolites were characterized by PLS-DA and VIP score (within 1.5 and 3.5). Among the top 10 metabolites, two volatile compounds; α-pinene and selina-3,7(11)-diene, can be used as chemical markers to determine the discrimination in five different *C. sativa* samples. However, volatile metabolites detected from the previous study were different from our study owing to the use of different cannabis cultivars and the number/amount of volatile compound detected (Zheng et al. 2014). Moreover, a previous study (Cicaloni et al. 2022) suggested that 8 non-volatile compounds can be used as chemical markers for discrimination in five different *C. sativa* samples. These non-volatile compounds are δ−9-cis-tetrahydrocannabinol, 2′-o-methylcajanone, ananolignan J, clovanemagnolol, kazinol F, cannabigerolic acid, monolenin, and labriformidin. The database of chemical markers could be further developed as chemical sensor for the simple recognition of single cannabis strains.

## Correlations of volatile compounds

**Fig. 5:**
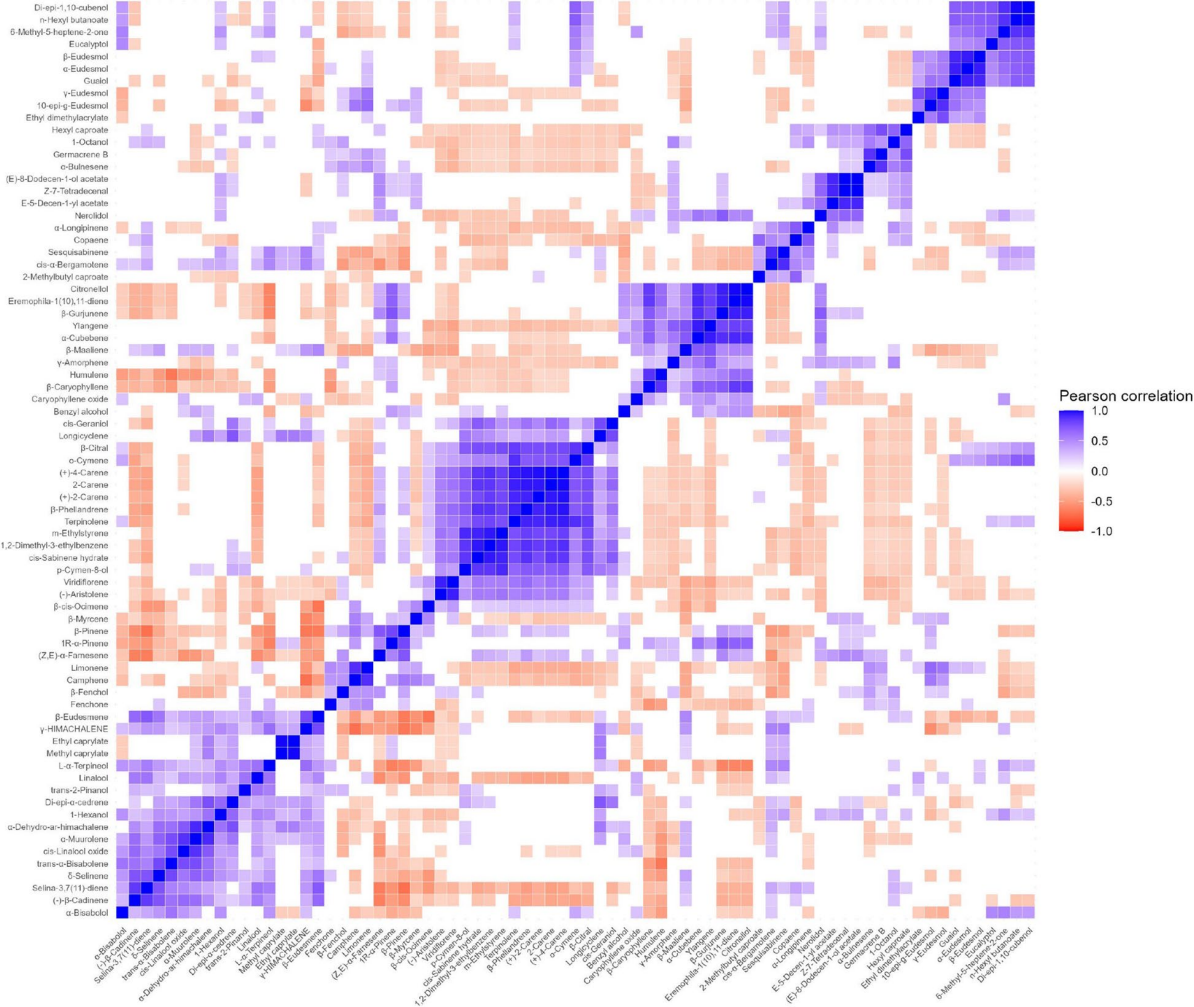
The overview correlation matrix based on Pearson Correlation Coefficient among 75 identifiable compounds

**Fig. 6:**
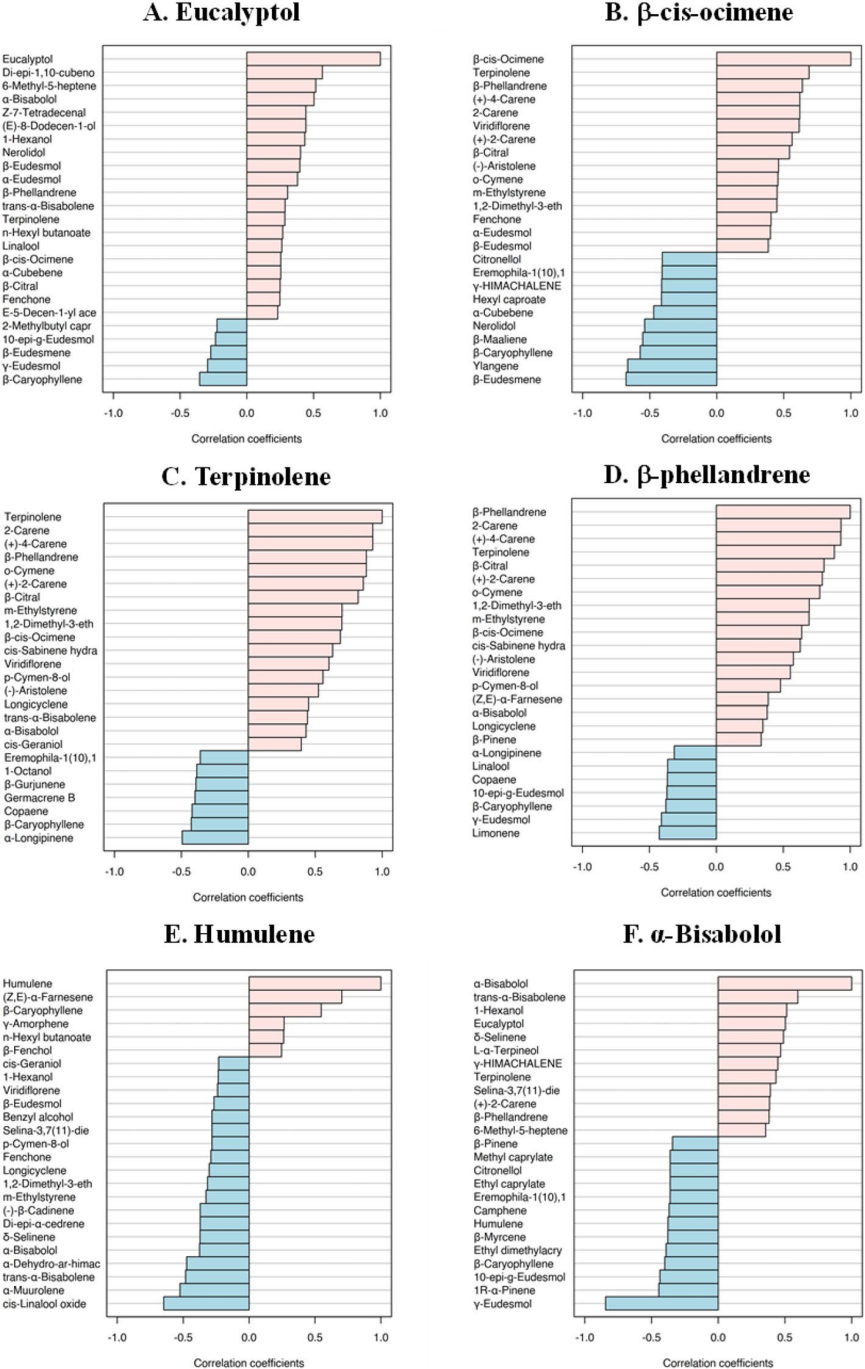
Bar plot bar plot based on Pearson Correlation Coefficient of (**A**) eucalyptol, (**B**) β-cis- ocimene, (**C**) terpinolene, (**D**) β-phellandrene, (**E**) humulene and (**F**) α-bisabolol, respectively

The relationships between 75 volatile compounds in 19 dried cannabis flowers were also investigated using Pearson’s correlation coefficient (r). This value was used as an evaluation index for prediction the correlation between two duplicate variables. Generally, *r* value was within the range of − 1 to 1. The closer the r is to 1, meaning the stronger positive correlation, while the closer the r is to − 1, indicating the stronger negative correlation. Basically, highly positively correlated volatiles were grouped in the same cluster, and compounds in distant clusters tend to show negative correlations. The overview correlation coefficient among variables within 75 identifiable compounds was shown in Fig. 5. The colored boxes of blue, red and white represent positive, negative and non-significant correlations, respectively. This study is especially described the top 20 correlated compounds which can be used as marker compounds to differentiate cannabis flower samples relied on the result of PLS-DA and VIP analysis as displayed in bar plot based on Pearson’s correlation coefficient (Fig. 6A and F).

Eucalyptol, β-cis- ocimene, terpinolene, β-phellandrene, humulene and α-bisabolol are some examples used to explain the volatile correlation. According to Fig. 6A, eucalyptol, found in low % area normalization in SK and WC (as can be seen in Fig. 2C), showed a strong inverse correlation with β-caryophyllene, showing high %area normalization in SK and WC. Moreover, eucalyptol displayed an inverse correlation trend with other compounds such as 2-methylbutyl caproate, 10-epi-γ-eudesmol, β-eudesmene and γ-eudesmol. Eucalyptol showed a positive correlation with terpinolene and linalool which are both highly detected in SK and WC. Figure 6B and C showed that terpinolene shared a positive correlation with β-cis-ocimene (showing highly detected in GDP, JH and GCP) as well as β-phellandrene (showing highly detected in WW, JH and BB). Terpinolene revealed a negative correlation with many other compounds; for instance, eremophila-1(10),11-diene, copaene, germacrene B and 1-octanol (Fig. 6C). The two latter compounds are characterized by low level in GCP. Humulene, characterized by high level, especially in FD, shared a similar trend with β-caryophyllene (with in agreement with a previous study (Cicaloni et al. 2022), (Z, E)-α-farnesene and γ-amorphene (Fig. 6E). Conversely, humulene showed inversely correlated with selina-3,7(11)-diene, showing low level in FD. According to Fig. 6F, α-Bisabolol was directly correlated with trans-α-bisabolene, L-α-terpineol and selina-3,7(11)-diene, but it was inversely correlated with β-pinene, β-myrcene, camphene and 1R- α-pinene. The 14 remaining correlated compounds; (+)−2-carene, o-cymene, γ-eudesmol, 1,2-dimethyl-3-ethylbenzene, α-longipinene, m-ethylstyrene, 10-epi-γ-eudesmol, γ-amorphene, (+)−4-carene, cis-geraniol, p-cymen-8-ol, 2-carene, β-citral and β-eudesmol were detailed in Fig. 3 (Supplementary material).

## Conclusions

Cannabis flower has a unique characteristic scent diversely among each cultivar. Its aroma is important for many applications; for example, aromatherapy, medical purposes, cannabis product manufacturing, and cannabis breeder. In this study, the chemical compositions of 19 different dried cannabis flower samples were successfully profiled with optimized HS-SPME-GC-MS. Seventy-five tentative compounds, including 9 chemical classes were identified. Sesquiterpenes and monoterpenes were predominant in all cannabis samples. Combining the use of chemometric tools, HCA and PCA successfully grouped the 19 cannabis cultivars into five main clusters based on their volatile chemotypes. For discovering chemical markers, PLS-DA and VIP scores were applied, identifying 20 markers for recognizing specific cultivars. Pearson’s correlation coefficient was an effective approach for studying the relationships among the 75 volatile compounds. However, the representativeness of the sample quantity should be carefully considered in future work. The overall database from this study will provide a scientific basis for identifying individual strains, verifying quality control, fulfilling commercial data on cluster analysis, and breeding programs of this plant in the future.

## Supplementary Information

Supplementary Material 1.

## Data Availability

The datasets performed and/or analyzed during the current study available from the corresponding author on reasonable request.
