# Supplementary material for: Chemical profiling and clustering of various dried cannabis flowers revealed by volatilomics and chemometric processing
Source: J Cannabis Res. 2024 Dec 6;6:41. doi: 10.1186/s42238-024-00252-w (PMC11622677; doi:10.1186/s42238-024-00252-w)
Supplement: Supplementary file 1 — Supplementary Material 1. [file 42238_2024_252_MOESM1_ESM.docx]

**Supplementary material**

**Chemical profiling and clustering of various dried cannabis flowers revealed by volatilomics and chemometric processing**

Pannipa Janta ^a,b^ and Sornkanok Vimolmangkang ^a,b,c,d*^

*^a^ Department of Pharmacognosy and Pharmaceutical Botany, Faculty of Pharmaceutical Sciences, Chulalongkorn University, Bangkok 10330, Thailand*

*^b^* *Phyto Analytica Testing Laboratory, Faculty of Pharmaceutical Sciences, Chulalongkorn University, Bangkok 10330, Thailand*

*^c^ Center of Excellence in Plant-Produced Pharmaceuticals, Faculty of Pharmaceutical Sciences, Chulalongkorn University, Bangkok 10330, Thailand*

*^d^ Research Cluster for Cannabis and its Natural Substances, Chulalongkorn University, Bangkok 10330, Thailand*

* Corresponding author. Department of Pharmacognosy and Pharmaceutical Botany,

Faculty of Pharmaceutical Sciences, Chulalongkorn University, Bangkok, Thailand. Tel.:6622188358.

*E-mail address:* sornkanok.v@pharm.chula.ac.th (S. Vimolmangkang).

**Table S1.** The 19 dried cannabis flowers and their commercial data on feelings, aromas, and cannabinoid levels.

| ***Cultivar*** | **feelings** | **aromas** | **%THC** | **%CBD** | **%CBG** |
| --- | --- | --- | --- | --- | --- |
| ***C. indica*** | | | | | |
| Grand Daddy Purple (GDP)* | Sleepy,  Hungry,  Relaxed | Grape, Berry, Sweet | 17 | 0 | No data |
| Skywalker OG  (SW-OG)* | Sleepy,  Relaxed,  Tingly | Spicy/herbal, pungent, earthy | 20 | No data | 1 |
| Northen Light  (NL)* | Sleepy,  Hungry,  relaxed | Pine, Spicy/herbal, earthy | 18 | No data | 1 |
| Wedding Cake  (WC)* | Relaxed,  Aroused,  hungry | Vanilla, pepper, sweet | 24 | No data | 1 |
| White Widow  (WW)* | Energetic,  Talkative,  uplifted | Woody, Spicy/herbal, earthy | 15 | No data | 1 |
| Purple Punch  (PP)* | Sleepy,  Relaxed,  Hungry | Grape, Berry, blueberry | 18 | 0 | No data |
| Pure Michigan (PM)* | Aroused, Relaxed, Sleepy | Chestnut, ammonia, lime | 29 | No data | 1 |
| Geta Fix (GF)**** | Euphoric, Relax, Tingly | Diesel, Fruity | 28 | No data | No data |
| ***C. sativa*** | | | | | |
| Jack Herer  (JH)* | Energetic,  Creative,  Uplifted | Pine, Spicy/herbal, woody | 18 | No data | 1 |
| Bruce Banner  (BB)* | Creative,  Energetic, euphoric | Strawberry, diesel, tea | 21 | No data | 1 |
| Green Crack Punch (GCP)** | Calming,  Euphoric | Citrus, Fruity | 18 | Low | No data |
| Amnesia Haze (AH)* | Energetic,  Talkative,  giggly | Lemon, citrus, lime | 19 | No data | 1 |
| Super Silver Haze (SH)* | Energetic,  Uplifted,  Creative | Spicy/herbal, citrus, skunk | 21 | No data | 1 |
| Skunk Haze (SK)* | Focused,  Relaxed,  Talkative | Spicy/herbal, skunk, pungent | 13 | No data | 1 |
| Banana Glue (BG)* | Euphoric, relaxed,  giggly | Earthy, woody, pine | 19 | 0 | No data |
| ***C. Hybrid*** | | | | | |
| Frisian Duck  (FD)* | Hungry, talkative,  Giggly | Spicy/herbal, pine, sweet | 15***** | 1***** | No data |
| Dulce de Fresa (DDF)*** | mood enhancement,  creative  relaxed | Sweet, Fruity | 28.66 | 0.07 | No data |
| Critical Purple Kush (CPK)* | Happy,  Sleepy, relaxed | Plum, chestnut, citrus | 13 | No data | 1 |

Commercial data on feelings and aromas of Hang Over G (HOG) is not available.

* Data from [www.leafly.com](http://www.leafly.com)

** Data from <http://www.royalqueenseeds.com>

*** Data from <https://herbalsolutions420.com/menu/?dtche%5Bproduct%5D=dulce-de-fresa-society-c>

**** Data from https://nasha-genetics.com/product/getafix/

***** Data from <https://weed.review/strains/frisian-duck/>

**Table S2. The figures-of-merit of each sample, analyzed in 5 replicates.**

| **Dried cannabis flower** | **Average total peak area**  **(*n* = 5)** | **%RSD** | **Average total peak height**  **(*n* = 5)** | **%RSD** | **Average peak width**  **(*n* = 5)** | **%RSD** |
| --- | --- | --- | --- | --- | --- | --- |
| Grand Daddy Purple (GDP) | 38159987204 | 4.8 | 4888116193 | 3.9 | 0.23 | 2.8 |
| Skywalker OG (SW-OG) | 52079455183 | 7.0 | 7489233731 | 5.5 | 0.19 | 2.1 |
| Northen Light (NL) | 68773410179 | 5.7 | 10345570328 | 5.0 | 0.20 | 0.8 |
| Wedding Cake (WC) | 49470931689 | 6.3 | 8114078178 | 5.4 | 0.19 | 1.8 |
| White Widow (WW) | 49053749145 | 6.4 | 7665135706 | 4.2 | 0.20 | 2.7 |
| Purple Punch (PP) | 33697723429 | 3.4 | 5509016048 | 2.8 | 0.20 | 1.4 |
| Pure Michigan (PM) | 48761642302 | 8.8 | 6122624527 | 7.0 | 0.23 | 2.6 |
| Geta Fix (GF) | 45844251212 | 7.2 | 6158732805 | 6.5 | 0.21 | 2.6 |
| Jack Herer (JH) | 52061941676 | 9.1 | 8027732711 | 8.3 | 0.20 | 1.1 |
| Bruce Banner (BB) | 47656602324 | 8.2 | 7812897499 | 8.2 | 0.19 | 2.2 |
| Green Crack Punch (GCP) | 55619104582 | 4.0 | 7403815683 | 4.1 | 0.24 | 4.9 |
| Amnesia Haze (AH) | 36057188593 | 3.5 | 5033556958 | 2.4 | 0.20 | 1.2 |
| Super Silver Haze (SH) | 30001259113 | 6.1 | 4920539142 | 5.4 | 0.21 | 2.1 |
| Skunk Haze (SK) | 18709493822 | 2.1 | 3456824318 | 2.3 | 0.19 | 1.9 |
| Banana Glue (BG) | 44575195943 | 6.6 | 6113502366 | 3.8 | 0.21 | 2.5 |
| Frisian Duck (FD) | 60555415581 | 2.9 | 6456806487 | 2.2 | 0.26 | 6.9 |
| Dulce de Fresa (DDF) | 38262868262 | 11.0 | 5604166928 | 8.8 | 0.22 | 1.3 |
| Critical Purple Kush (CPK) | 37303373759 | 3.5 | 5916183890 | 2.2 | 0.15 | 2.1 |
| Hang Over G (HOG) | 35005699774 | 4.0 | 4674979175 | 3.2 | 0.22 | 1.8 |

**Table S3.** The variable importance in projection (VIP) scores of 75 variable compounds within the partial least-squares discriminate analysis (PLS-DA) model.

| **Variable** | **VIP scores** |
| --- | --- |
| Eucalyptol | 3.128 |
| (+)-2-Carene | 3.082 |
| o-Cymene | 2.258 |
| Terpinolene | 2.114 |
| γ-Eudesmol | 2.085 |
| α-Bisabolol | 1.903 |
| 1,2-Dimethyl-3-ethylbenzene | 1.806 |
| α-Longipinene | 1.727 |
| m-Ethylstyrene | 1.554 |
| β-cis-Ocimene | 1.474 |
| 10-epi-γ-Eudesmol | 1.366 |
| β-Phellandrene | 1.358 |
| Humulene | 1.274 |
| γ-Amorphene | 1.243 |
| (+)-4-Carene | 1.222 |
| cis-Geraniol | 1.220 |
| p-Cymen-8-ol | 1.217 |
| 2-Carene | 1.197 |
| β-Citral | 1.051 |
| β-Eudesmol | 1.051 |
| cis-Sabinene hydrate | 0.909 |
| Longicyclene | 0.906 |
| β-Eudesmol | 0.893 |
| α-Muurolene | 0.869 |
| n-Hexyl butanoate | 0.853 |
| trans-α-Bisabolene | 0.840 |
| Ethyl dimethylacrylate | 0.833 |
| α-Dehydro-ar-himachalene | 0.803 |
| 1-Hexanol | 0.775 |
| (-)-Aristolene | 0.755 |
| cis-Linalool oxide | 0.736 |
| 1-Octanol | 0.694 |
| (Z,E)-α-Farnesene | 0.608 |
| E-5-Decen-1-yl acetate | 0.585 |
| 6-Methyl-5-heptene-2-one | 0.581 |
| Di-epi-α-cedrene | 0.564 |
| Benzyl alcohol | 0.562 |
| β-Gurjunene | 0.553 |
| trans-2-Pinanol | 0.547 |
| Viridiflorene | 0.542 |
| α-Bulnesene | 0.514 |
| Selina-3,7(11)-diene | 0.393 |
| L-α-Terpineol | 0.361 |
| cis-α-Bergamotene | 0.307 |
| Fenchone | 0.297 |
| Copaene | 0.273 |
| β-Caryophyllene | 0.273 |
| Methyl caprylate | 0.265 |
| Di-epi-1,10-cubenol | 0.261 |
| Ylangene | 0.260 |
| Linalool | 0.258 |
| Ethyl caprylate | 0.254 |
| Nerolidol | 0.224 |
| δ-Selinene | 0.205 |
| Germacrene B | 0.196 |
| Guaiol | 0.191 |
| (-)-β-Cadinene | 0.185 |
| Citronellol | 0.183 |
| Eremophila-1(10),11-diene | 0.169 |
| Sesquisabinene | 0.167 |
| β-Eudesmene | 0.148 |
| β-Myrcene | 0.146 |
| β-Maaliene | 0.137 |
| Caryophyllene oxide | 0.113 |
| β-Fenchol | 0.101 |
| (E)-8-Dodecen-1-ol acetate | 0.091 |
| Z-7-Tetradecenal | 0.090 |
| 1R-α-Pinene | 0.074 |
| 2-Methylbutyl caproate | 0.067 |
| α-Cubebene | 0.061 |
| Limonene | 0.059 |
| Camphene | 0.033 |
| γ-HIMACHALENE | 0.015 |
| Hexyl caproate | 0.007 |
| β-Pinene | 0.006 |

**Figure S1**. TIC of a representative cannabis flower sample in each cluster.


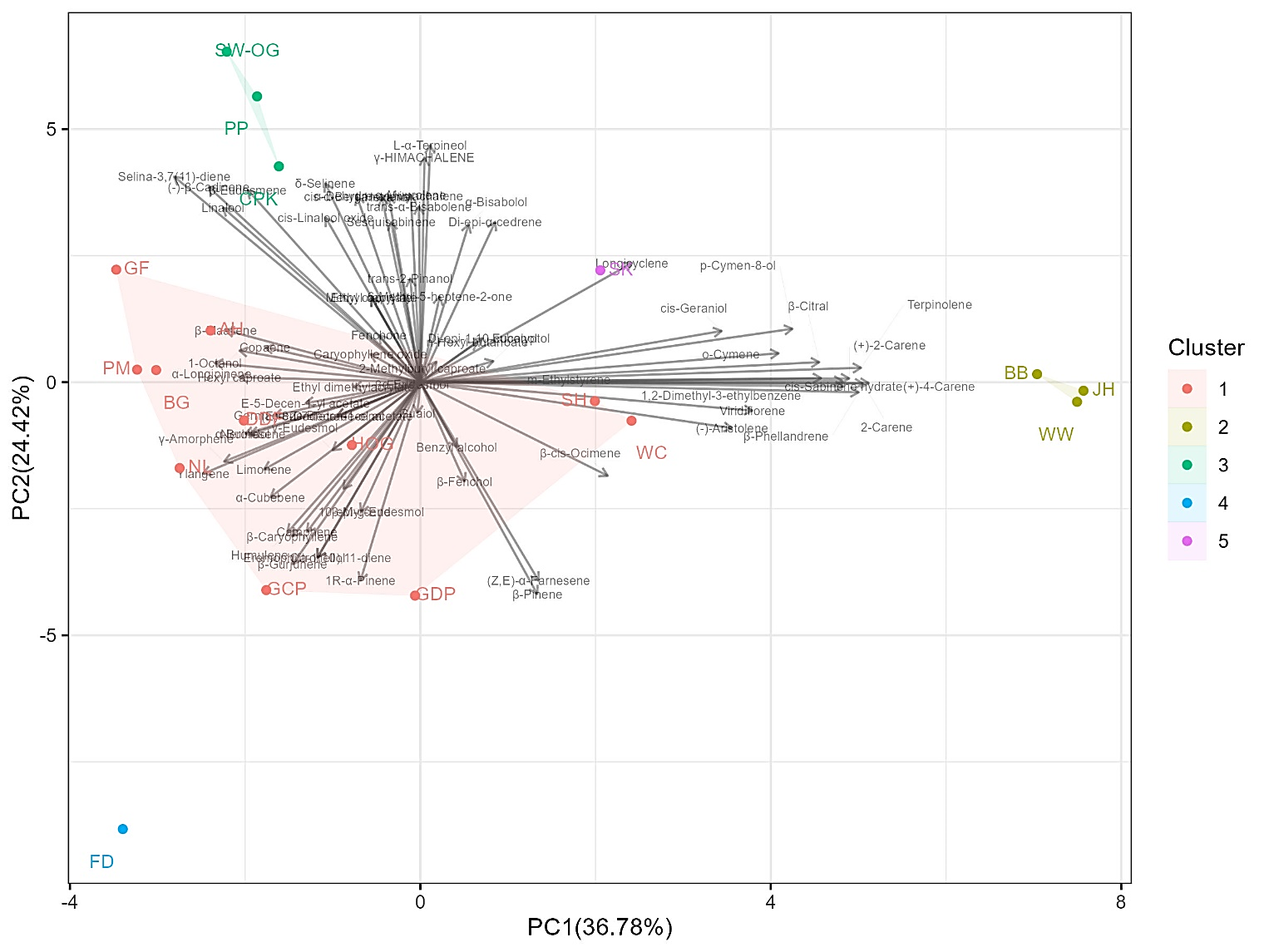


**Figure S2**. PCA biplot, showing main volatile compounds found in each cluster.

**Figure S3.** Bar plot based on Pearson Correlation Coefficient of 14 remaining correlated compounds; (1) (+)-2-carene, (2) o-cymene, (3) γ-eudesmol, (4) 1,2-dimethyl-3-ethylbenzene, (5) α-longipinene, (6) m-ethylstyrene,(7) 10-epi-γ-eudesmol, (8) γ-amorphene, (9) (+)-4-carene, (10) cis-geraniol, (11) p-cymen-8-ol, (12) 2-carene, (13) β-citral, and (14) β-eudesmol, respectively.
